# A Genetic Screen for Investigating the Human Lysosomal CystineTransporter, Cystinosin

**DOI:** 10.1038/s41598-018-21483-x

**Published:** 2018-02-21

**Authors:** Anup Arunrao Deshpande, Anuj Shukla, Anand Kumar Bachhawat

**Affiliations:** Indian Institute of Science and Education Research Mohali, Sector 81, Knowledge City, SAS Nagar, Punjab, India

**Keywords:** Biochemistry, Membrane trafficking, Genetics

## Abstract

Cystinosin, a lysosomal transporter is involved in the efflux of cystine from the lysosome to the cytosol. Mutations in the human cystinosin gene (*CTNS*) cause cystinosis, a recessive autosomal disorder. Studies on cystinosin have been limited by the absence of a robust genetic screen. In the present study we have developed a dual strategy for evaluating cystinosin function that is amenable to rapid genetic analysis. We show that human cystinosin expressed in this yeast confers growth on cystine when the protein is mistargeted to the plasma membrane by the deletion of the C-terminal targeting signal, GYQDL. We also screened a vacuolar protein sorting deletion library, and subsequently created multiple *vps* deletion mutants for kinetic studies. The double deletion, *vps1*Δ*vps17*Δ, greatly enhanced uptake. This enabled validation by kinetic studies, including first studies on the WT CTNS protein (that contained the GYQDL motif). Using this screen we isolated several gain of function mutants, G131S/D, G309S/D, A137V, G197R, S270T, L274F and S312N showing enhanced growth on low concentrations of cystine. Kinetic analysis yielded insights into the role of the residues (including one of the patient mutations, G197R). The results indicate that the screen could be effectively used for interrogating and understanding the *CTNS* protein.

## Introduction

Cystinosin (encoded by the *CTNS* gene) is a member of the PQ-loop family of transporters, and is involved in the efflux of cystine from the lysosome to the cytosol. Mutations in the human cystinosin gene (*CTNS*) have been shown to be responsible for an autosomal monogenic recessive disorder, cystinosis. Defective functioning of cystinosin in humans leads to excessive accumulation of cystine in the lysosome^1^. The pathophysiology of the disease is however not fully understood^2–4^. The current treatment of cystinosis uses cysteamine, which when taken up in the cell, moves to the lysosome, and forms cysteine-cysteamine mixed disulphides, that lead to the depletion of cystine since this disulphide can be effluxed out of the lysosome through a different lysosomal PQ-loop efflux pump, the *PQLC2* transporter^5^. In the absence of cysteamine therapy, and in severe cases, with age, progressive renal injury is known to occur leading to end state renal disease^6,7^.

Despite the discovery of cystinosin almost 2 decades back, surprisingly little is known about the protein. As cystinosin is a lysosomal transporter, some of the targeting signals have been identified. These include the C-terminal tyrosine based lysosomal targeting motif GYDQL and a second putative motif based in the third cytoplasmic loop, YFPQA. Deletion of both of these motifs leads to complete re-localization of *CTNS* to plasma membrane^8^. However, deletion of YFPQA also leads to a non-functional protein, and residues in this region could not be pinpointed for their involvement, suggesting that the YFPQA stretch may not in fact be a true targeting motif. Thus only GYDQL has been identified as a targeting motif, but it is likely that other residues may be involved considering that deletion of GYDQL leads to only partial mislocalization. However, these additional targeting motifs have not been adequately explored. The molecular function of cystinosin has been characterized by redirecting it to the plasma membrane by deleting the first lysosomal sorting motif GYDQL present at the C-terminal region^9^. Several patient mutations have been evaluated in mammalian cells or Xenopus oocytes using this approach. Cystinosin, a proton/cystine symporter also has a duplicated motif termed “PQ Loop” and the residue D305 from PQ loop 2 has been found to be crucial for proton translocation^10^. Despite these studies, the mechanism of cystine transport by cystinosin is still not fully understood. A three dimensional model is still not available, and a detailed mechanistic understanding of substrate binding and translocation by cystinosin is still lacking. In the absence of structural information, genetic approaches can provide excellent insights into function of transporters. In the case of cystinosin the only biochemical assays available are either radioactive uptake in mammalian cell lines or transport studies using Xenopus oocytes. Genetic studies have been hampered by the absence of a simple genetic screen and assay.

In this manuscript we describe the development, evaluation and power of a yeast based screen for the cystinosin transporter. The genetic assay in yeast has exploited the inability of the yeast *Saccharomyces cerevisiae* to transport cystine. Thus mistargeting of human cystinosin lacking the C-terminal lysosomal trafficking GYDQL motif to the plasma membrane of yeast enabled yeast to grow on cystine as a sulphur source. The precise residues Y and L involved in the mammalian system were also critical in the yeast system. As radioactive uptake levels were suboptimal, we evaluated and exploited vacuolar protein missorting mutants of yeasts to increase cell surface expression of this transporter, and this approach successfully enabled us to carry out more quantitative evaluations using radioactive uptake of cystine in whole cells. The kinetic parameters of WT CTNS (that included the GYDQL motif) were determined for the first time using this system. CTNS-Δgydql had kinetic parameters similar to what has been determined in the Xenopus and cell line models validating the assay. This functional growth-based screen enabled analysis of loss-of-function patient mutations, and the isolation of new gain-of-function mutants. The functional characterization of these latter mutants revealed two classes of mutants. Mutants in one of these classes were leading to altered trafficking. The studies have thus yielded new insights into the cystinosin protein and the screen should further serve as a useful aid for subsequent investigations with the *CTNS* protein.

## Results

### Deletion of the C-terminal lysosomal targeting signal of cystinosin ‘GYDQL’, leads to mis-targeting of *CTNS* to the plasma membrane in yeast and growth on cystine as a sulphur source

**Figure 1:**
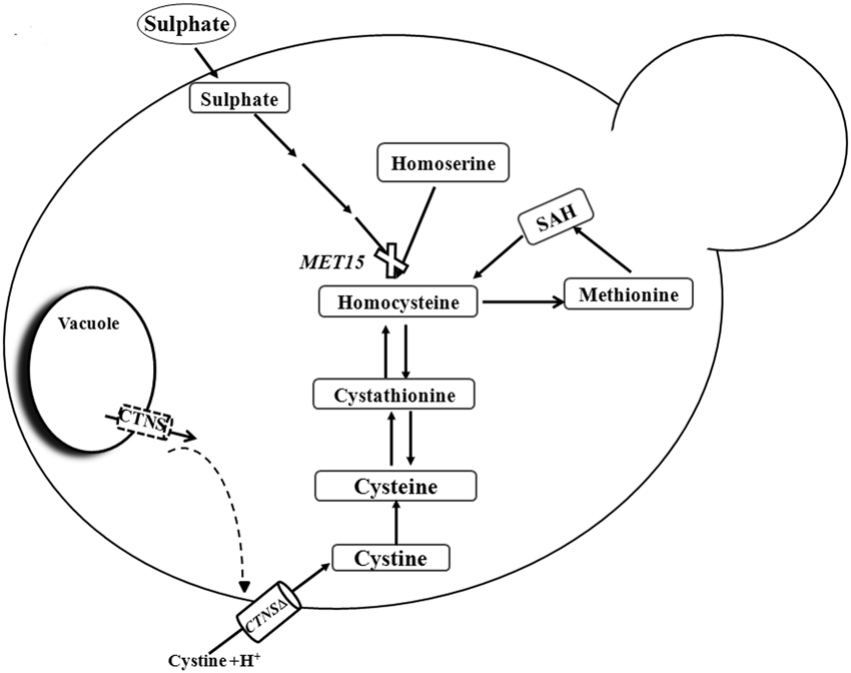
Schematic figure showing how a heterologously expressed CTNS and CTNS-Δgydql could confer growth on cystine in an *S*. *cerevisiae met15*Δ strain. Heterologous expression of CTNS in *S*. *cerevisiae* is expected to cause its intracellular retention, whereas expression of CTNS**-**Δgydql (lacking C-terminal lysosomal targeting motif) possibly misdirects the protein to the plasma membrane leading to cystine intake from the extracellular milleu.

The yeast *S*. *cerevisiae* lacks the ability to utilize cystine as a sulphur source owing to lack of a plasma membrane based cystine transporter. Thus a *S*. *cerevisiae met15*Δ strain which is an organic sulphur auxotroph is unable to grow on inorganic sulphur but can grow on organic sulphur sources such as methionine, cysteine, glutathione and homocysteine, but not cystine^11^. However if a plasma membrane based cystine transporter is heterologously expressed in yeast, it can permit the growth on cystine^12^. We have used this property to examine if human cystinosin can confer on *S*. *cerevisiae* the ability to grow on cystine (Fig. 1).

**Figure 2:**
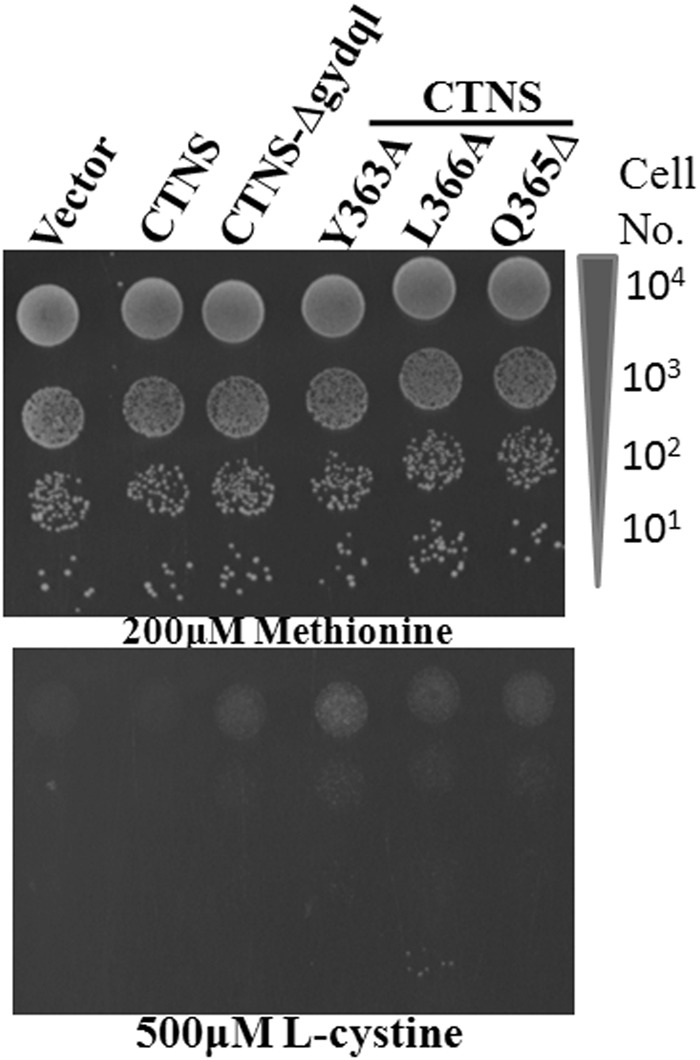
Growth of CTNS mutants defective in lysosomal targeting. The *S*. *cerevisiae met15Δ yct1Δ* strain (ABC 1580) was transformed with wild-type CTNS and the mutant of defective in lysosomal trafficking (*CTNS*-Δgydql) expressed under TEF promoter, along with, Y363A and L366A mutants, and vector control, examined by serial dilution spotting on minimal media containing 200 µM Methionine & 500 µM L-cystine. The photographs were taken after 2 days of incubation at 30 °C. The experiment was repeated with two independent transformations.

CTNS WT and CTNS-Δgydql were expressed from a TEF promoter and transformed into *S*. *cerevisiae met15Δ* strain. In many experiments we additionally used a *yct1Δ* background that was deficient in the high affinity cysteine transporter and minimized contaminating influences of cysteine in cystine preparations. The transformants were spotted on methionine and on 500 µM cystine plates. The CTNS-Δgydql constructs were able to confer growth in cystine after 3 days of incubation, but not the WT construct (Fig. 2). This suggests that the GYDQL motif was also functioning to retain the protein intracellularly in yeast and that deletion of the motif led to at least partial mistargeting to the plasma membrane.

**Figure 3:**
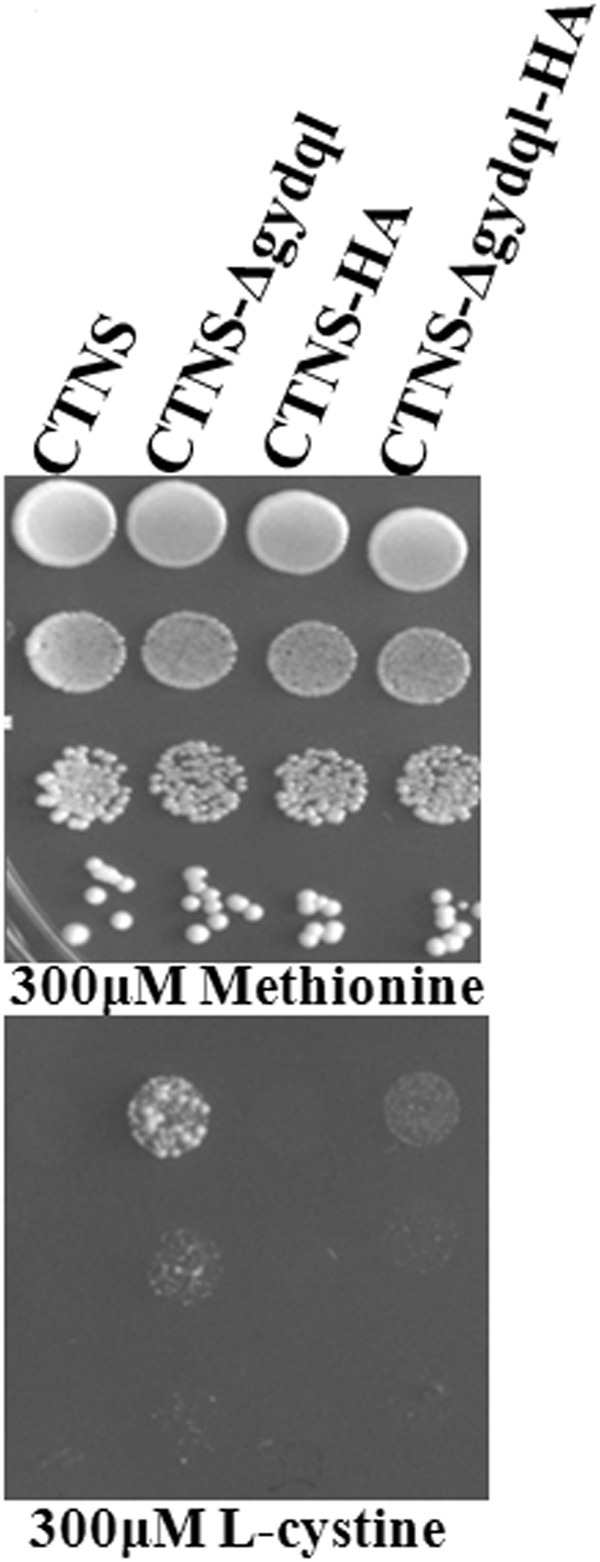
Functional analysis of HA-tagged CTNS, CTNS-Δgydql constructs. *S*. *cerevisiae* ABC 1580 (*met15∆yct1∆*) transformed with plasmids bearing HA epitope tagged CTNS, CTNS-Δgydql constructs expressed from TEF promoter and examined by dilution spotting on minimal media containing 200 µM Methionine, & 300 µM L-cystine.The photographs were taken after 3.5 days of incubation at 30 °C. The experiment was repeated with two independent transformations.

We also generated a C-terminally HA tagged construct for CTNS-Δgydql. Comparative analysis of these constructs with untagged versions by functional complementation of these constructs shows a decreased functionality in the case of C- terminally HA tagged-Δgydql construct (Fig. 3). Since C-terminal residues are known to be involved in the trafficking to lysosome, inclusion of additional residues downstream to the C-terminus was probably affecting its localization. Thus majority of the experiments were carried out using an untagged cystinosin constructs with the exception of localization studies where C-terminally HA tagged constructs were used. Commercially available anti-cystinosin antibodies have been reported to be unreliable for the understanding the subcellular distribution of the protein^13^.

To identify the principal motif targeting *CTNS* to the vacuole in yeasts in a more unbiased approached, we attempted to isolate random mutations in *CTNS* that would mislocalize it to the plasma membrane. The plasmid p416TEF-CTNS, an expression plasmid for *CTNS* in yeast was subjected to *in vitro* hydroxyl amine mutagenesis and transformed in *S*. *cerevisiae met15*Δ *yct1*Δ strain. The mutagenesis yielded one mutant. Sequencing analysis revealed the presence of a mutation in the C-terminal tyrosine motif GYDQL, where Q was converted to a nonsense codon (C1093T: Q365Δ) leading to premature truncation of the protein at the GYDQL motif. The mutagenesis strategy in yeast further confirmed the role of the C-terminal motif as being an important motif in vacuolar targeting of this protein to the vacuole in yeast (Fig. 2).

### The role of the “Y” and “L” residues in the GYQDL motif in targeting *CTNS* to the lysosome is conserved in yeast

In the GYDQL lysosomal targeting motif, tyrosine Y363 and leucine L366 are the key residues and deletion of either leads to partial mis-localization of CTNS to plasma membrane in mammalian cells^8^. To see if the same residues are likely to be involved in retaining *CTNS* intracellularly in yeast, we created Y363A and L366A mutations and examined the ability of the constructs to confer growth on cystine. Interestingly, we observed that both mutants allowed growth on cystine, comparable to the CTNS-Δgydql suggesting that these mutants were being mistargeted to the plasma membrane (Fig. 2). These findings also suggest that for *CTNS* trafficking in yeast, similar membrane trafficking adaptors could be involved in yeast and mammalian cells.

### Evaluation of the yeast growth assay analysing loss of function patient mutation

**Figure 4:**
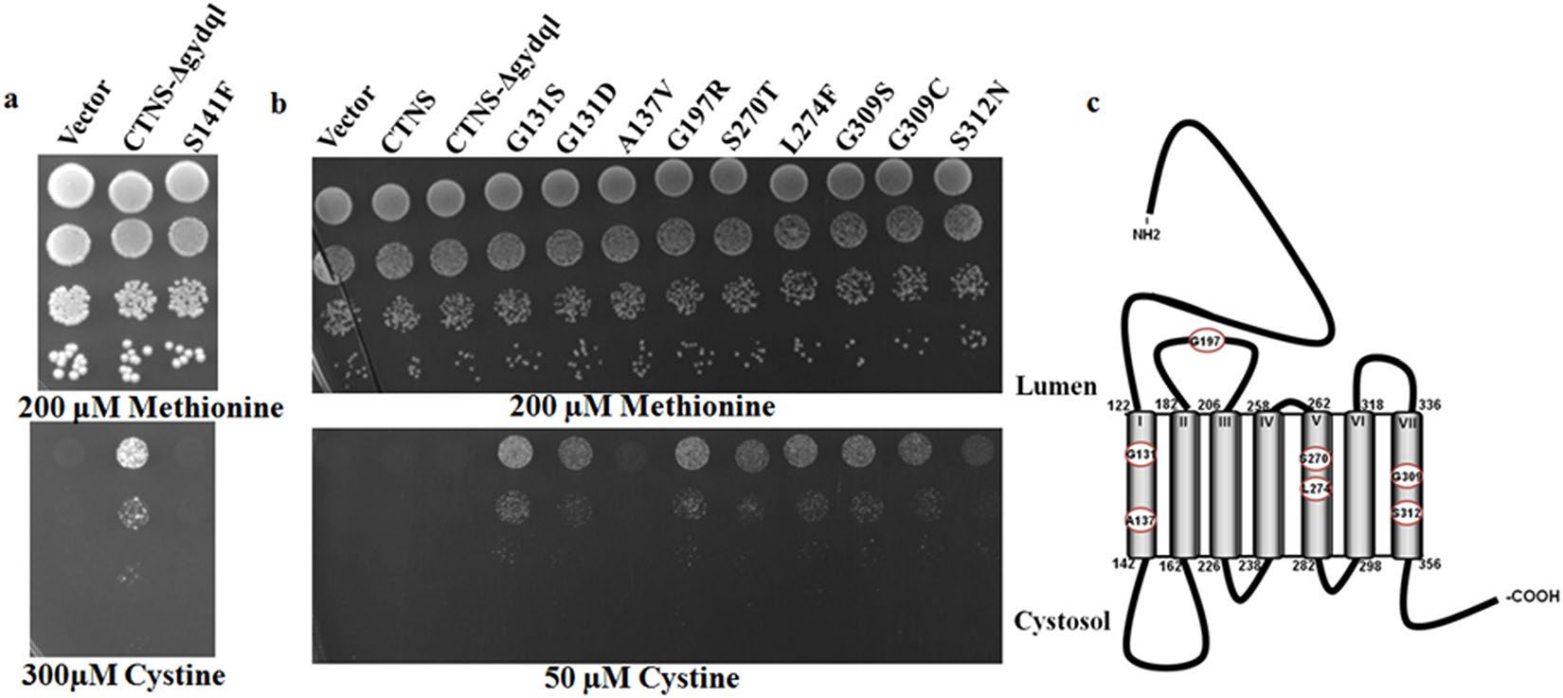
Functional analysis of CTNS mutants. (**a**) Functional analysis of loss of function patient mutation S141F. Transformant’s were serial diluted and spotted on minimal media containing 200 µM Methionine, & different concentration of cystine as described in experimental procedures. The photographs were taken after 2–3 days of incubation at 30 °C. The experiment was repeated with three independent transformations. (**b**) Gain of function mutants The *S*. *cerevisiae met15*Δ*yct1*Δ strain (ABC 1580) was separately transformed with WT CTNS, CTNS**-**Δgydql, p416-TEF (control vector) and the different gain of function mutants expressed under the TEF promoter. (**c**) Gain-of-function mutations mapped on the *CTNS* topology.

In a recent study we identified S141F as a frequently occurring mutation among Indian patients affected with infantile cystinosis^14^. S141F mutation is known to cause a severe defect in the functionality of the transporter (2 ± 5.3% of WT cystinosin activity)^15^. In order to evaluate the effect of S141F mutation on *CTNS* functionality using the yeast based assay, we reconstructed the S141F mutation in CTNS-Δgydql in the yeast expression plasmid. Transformation of the CTNS*-*Δgydql S141F mutant plasmid in *S*. *cerevisiae met15*Δ*yct1*Δ failed to confer growth on the selection plate containing 300 µM cystine. The lack of complementation of the S141F mutation in the yeast assay demonstrated the usefulness of the yeast assay for rapidly and inexpensively evaluating the functionality of the protein in patient mutations (Fig. 4a).

### Isolation of gain-of-function mutants of CTNS-Δgydql showing the ability to grow on low concentrations of cystine

An important objective of developing such a growth based screen was to initiate a molecular genetic based analysis on the protein. To embark on this, we sought to isolate mutants of *CTNS* using this screen. The ‘wild-type’ CTNS-Δgydql construct was able to confer growth on cystine, but growth was slow, and could be observed only at higher concentrations (>300 µM cystine). We used this property to isolate mutants showing increased growth at lower concentrations of cystine in the medium. Such gain of function mutants would be expected to have either higher substrate affinity, higher substrate translocation or increased mistargeting to the plasma membrane. The p416TEF-CTNS-Δgydql construct was subjected to hydroxylamine mediated random mutagenesis and the purified mutagenized plasmid was transformed in *S*. *cerevisiae met15*Δ*yct1*Δ background strain. The transformants were selected on 50 µM cystine plates. Plasmids were isolated from 14 of the 24 transformants, purified in *E*. *coli* and retransformed into yeast to confirm the phenotype. Sequencing analysis of the purified plasmids revealed that out of total 14 independent mutants analyzed, 7 different mutations were identified in 5 different residues. These mutations were G131S, G131D, G197R, S270T, L274F, G309S and G309C (Fig. 4b). Among them, G131 residue in TMD1 was represented twice (G131D, G131S), while each G309C and G309S mutant in TMD6 occurred twice and L274 in TMD5 occurred 4 times (L274F). Two of the plasmids carried a mutation in T334S and V89M respectively which did not appear to have a significant gain of function phenotype and were not pursued further. Out of these G131, S270, L274, G309 were found to be in the putative trans-membrane domains (Fig. 4C, Table S4). Interestingly, most of the residues had a significant level of conservation (Fig. S1). Surprisingly some of these residues such as G197, S270 and G309 are also associated with different cystinotic patients^15–17^.

To obtain insights into how the isolated mutations were leading to increased functional activity of *CTNS*, and if the specific change was important, we created alanine mutations at a few of these gain of function site using site directed mutagenesis. Since most of the residues were in the TMDs, we chose to make the change to alanine, which would be tolerated within the TMD. These alanine mutants of *CTNS* (G131A, G197A, S270A, L274A, G309A) were transformed into the *S*. *cerevisiae* strain and compared for their functional activity with the wild type CTNS-Δgydql (pTEF-CTNS-Δgydql) using the growth assay on cystine plates (Fig. S2). Interestingly uptake of cystine analyzed by the growth assays using different concentration of cystine revealed that the alanine mutants of G131, G197, S270 and G309 retained gain-of- function property to a large extent. This indicated that the loss of the original conserved residue was critical. Only the L274A mutant however, showed differences with the L274F mutant.

### Radioactive uptake of cystine conferred by the CTNS-Δgydql containing transformants

Although the growth based screen for cystinosin function seemed suitable for isolating mutants and for evaluating non-functional patient mutations, a more quantitative comparison was necessary to characterize mutants with altered activity. In order to supplement the growth experiments by biochemical assays, radiolabelled ^14^C-cystine uptake measurements were carried out. However, surprisingly, no uptake was observed in CTNS-Δgydql expressing transformants even after incubation for prolonged period (up to 40 minutes) with radiolabelled cystine. In contrast the positive control, the *Candida glabrata* cystine transporter, *CgCYN1* showed significant uptake which is also reflected in the faster growth by *CgCYN1* as seen on plates (data not shown). Thus unlike the clear phenotypes on the plates, the uptake was too low to allow any comparisons of the mutants.

### Screening the vacuolar protein sorting deletion collection of yeast reveals mutants that show better growth on cystine

One possible explanation for the poor radiolabelled cystine uptake seen with the CTNS-Δgydql transformed cells was the poor surface expression due to only a partial mislocalization of the CTNS-Δgydql to the plasma membrane. Although this was adequate when growth was observed over 3–4 days it was inadequate for conferring cystine uptake. In mammalian cells also it has been observed that while deletion of the GYDQL motif led to some cell surface expression mislocalization of *CTNS*, a significant amount of the protein was still intracellular^18^.

**Figure 5:**
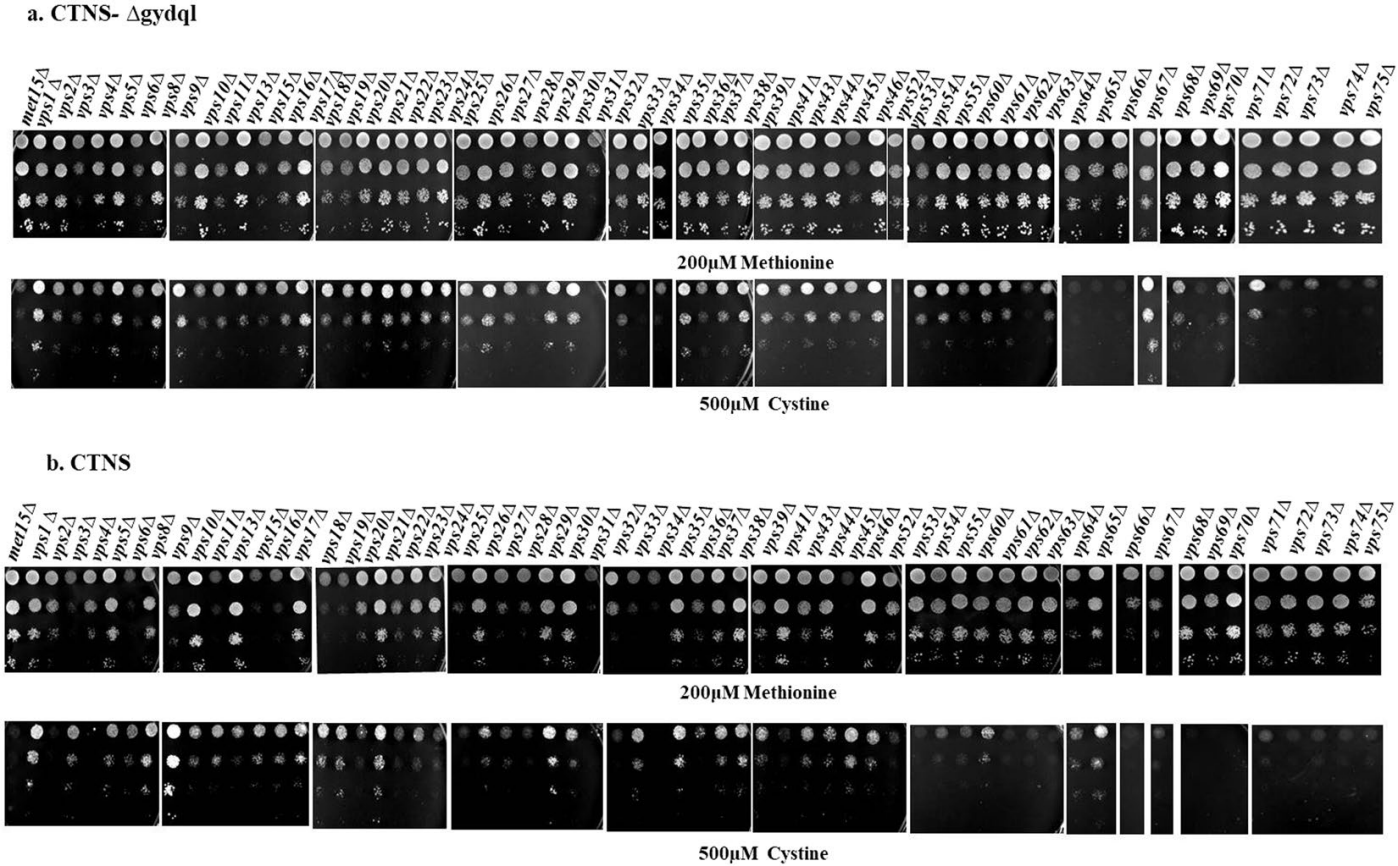
Functional analysis of CTNS and CTNS-Δgydql transformed in different *vps* mutant backgrounds. Different vacuolar protein sorting defective mutants (*met15Δ*:BY4741) transformed with (**a**) CTNS-Δgydql and (**b**) CTNS expressed under TEF promoter, and examined by dilution spotting on minimal media containing 200 µM Methionine, & 500 µM L-cystine expressed as described in experimental procedures. The photographs were taken after 3 days of incubation at 30 °C. The experiment was repeated with three independent transformations.

To address the problem of lower uptake it was important to maximize the amount of protein on the plasma membrane. Increasing the diversion of intracellular CTNS-Δgydql to the plasma membrane seemed the logical way to increase the amount of protein on the membrane. This might then be reflected in increased uptake of radiolabelled cystine. We decided to examine the different vacuolar protein sorting (*vps*) mutants^19–21^ to see if any of the mutants might lead to increased growth and cystine uptake. We procured the collection of *vps* deletion mutants from Euroscarf that includes mutants that affected both the CPY pathway and ALP pathway and transformed each of them with CTNS-Δgydql. Transformants were evaluated at 250 µM and 500 µM cystine (Supplementary Fig. S3). Compared to control, 10 *vps* mutants (*vps1*Δ, *vps8*Δ, *vps9*Δ, *vps35*Δ, *vps38*Δ, *vps39*Δ, *vps43*Δ, *vps46*Δ, *vps67*Δ, *vps71*Δ) showed better growth on cystine medium, while majority of the rest showed a moderate growth phenotype (Fig. 5a). *vps45*Δ which showed better growth with transformants, was itself slow growing and was therefore not included in our shortlist. We then evaluated if the CTNS wild type construct (containing the GYDQL motif) might also be able to confer growth in some of the *vps* mutant backgrounds (Supplementary Fig. S4). Interestingly, we observed that many of the *vps* mutant backgrounds allowed CTNS WT to confer growth on cystine medium (Fig. 5b). In particular we observed that the *vps1*Δ and the *vps9*Δ backgrounds in particular gave very consistent good growth.

Comparison of the deletion mutants in conferring growth on cystine medium with either CTNS or CTNS-Δgydql revealed that consistently better growth was observed with the *vps1*Δ mutant with both constructs and we decided to pursue with this genetic background further. *VPS1* is a dynamin related GTPase protein with promiscuous functions such as vacuolar trafficking, peroxisome fission and endocytosis^22,23^. *VPS1* participates in trafficking from TGN (trans-Golgi network) to endosome and TGN to vacuole protein transport and in endocytosis it facilitates directed invagination leading to effective scission. Deletion of *VPS1* leads to diversion of membrane traffic departing from the late Golgi normally destined for the prevacuolar compartment to the plasma membrane^24^. Since this deletion affects the endocytosis process, the protein might be retained on the cell surface for the longer duration, thereby increasing the protein levels on the plasma membrane at the given time interval. We also found that the vacuolar protein sorting mutants enabled even the WT CTNS protein (with the GYDQL motif intact) to grow on cystine and (Fig. 5b). We also used these genetic backgrounds to isolate mutants growing at very low concentrations of cystine. In addition to isolating the previously isolated mutants, L274F (3 times), G131D (1 time), G309S(3 times), G197R (3 times), we isolated 2 novel mutants A137V (1 time) and S312N (2 times) (Supplementary Table S4). However, these latter mutants did not show as strong a growth advantage when placed back in the WT background (Fig. 4b).

### Construction and evaluation of vps1 deletions carrying deletions in other mutant backgrounds to increase cell surface expression and enhanced growth on cystine*-vps1*Δ*vps17*Δ shows significant enhancement

**Figure 6:**
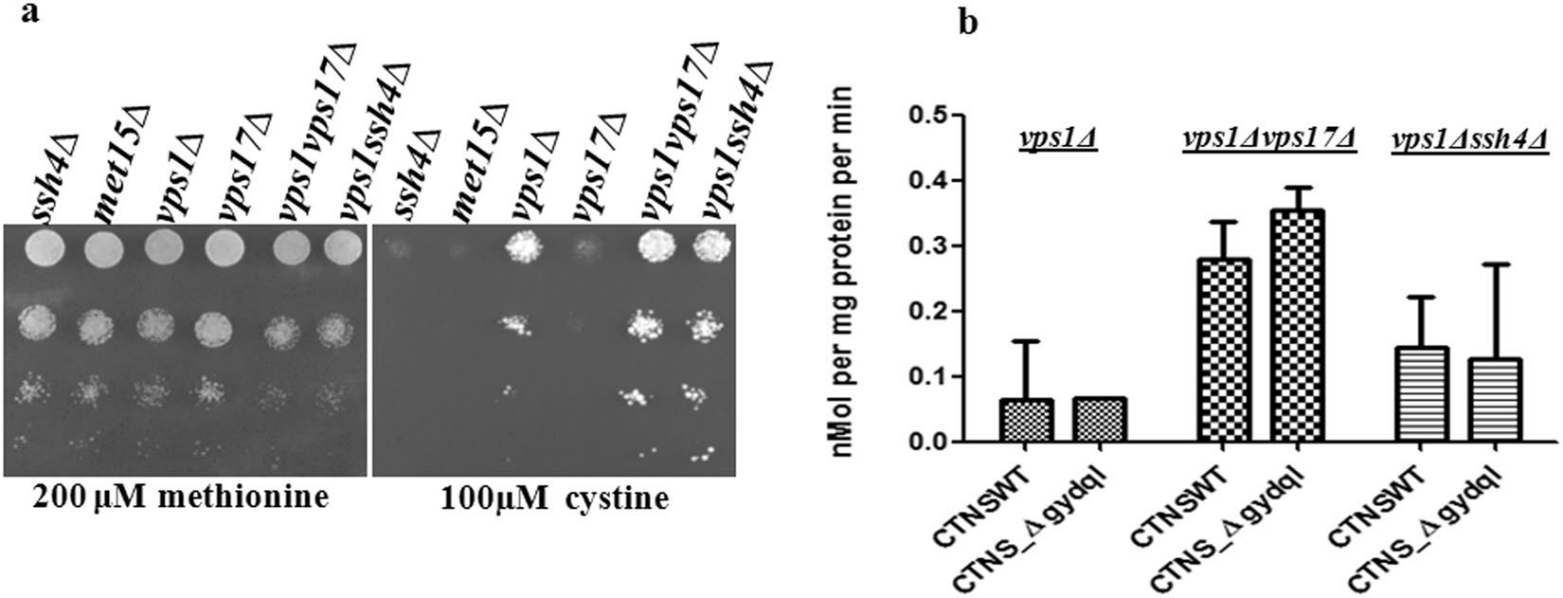
(**a**) Growth of CTNS-Δgydql transformed in different single delete and double delete *vps* mutants on medium containing cystine. Different vacuolar protein sorting defective mutants transformed with, *CTNS*-Δgydql expressed under TEF promoter, and examined by dilution spotting on minimal media containing 200 µM Methionine, & different concentration of cystine as described in experimental procedures. The photographs were taken after 2–3 days of incubation at 30 °C. The experiment was repeated with three independent transformations. (**b**) Comparison of uptake in *vps1*Δ, *vps1*Δ*vps17*Δ and *vps1*Δ*ssh4*Δ backgrounds. For quantification of functional activity of the CTNS constructs in different backgrounds, the initial rate of ^35^S-cystine uptake was measured in *S*. *cerevisiae vps1*Δ, *vps1*Δ*vps17*Δ and *vps1*Δ*ssh4*Δ transformed with CTNS-WT, CTNS-Δgydql in presence of 50 µM cystine. The cells were harvested at 10 and 20 min. intervals and results were plotted as nmol/mg protein/min after deducting the background uptake. The results represent the means of two different experiments, each in duplicate (±S.D., n = 4).

Although *vps1*Δ showed increased cell surface expression of the *CTNS* and CTNS-Δgydql, it still appeared inadequate for kinetic evaluations. As other *vps* mutants represent and participate in other parallel pathways for targeting of *CTNS* to the vacuole, we decided to create double deletions genes participating in both the ALP and CPY pathways that included genes participating in endocytosis and retrograde protein transport in a *vps1*Δ deletion background. We also included other gene deletion backgrounds that have shown to affect cell surface expression of other proteins. This includes the *SSH4* gene that encodes a specificity factor for *RSP5*, and is required for sorting of cargo proteins at the multivesicular bodies^3^. This gene was initially isolated as a high copy suppressor of *SHR3* deletion leading to increased amino acid permease at the cell membrane^6^. Other gene deletions that we included in this analysis was *DOA4*, a ubiquitin hydrolase that deubiuitinates intraluminal cargo proteins^25,26^, and *CLC1* which encodes the clathrin light chain^27^. In all a total of 9 double deletions were created in the *vps1*Δ background and involved the following gene deletions of *vps9*Δ, *vps10*Δ, *vps17*Δ, *vps27*Δ, *vps44*Δ, *clc1*Δ, *ssh4*Δ and *doa4*Δ. These double deletions were analysed for their ability to mistarget the CTNS and CTNS-Δgydql to the cell surface by means of growth on the minimal medium containing different concentrations of cystine (Supplementary Fig. S5). Significantly better growth was observed with the *vps1*Δ*vps17*Δ and the *vps1*Δ*ssh4*Δ mutants (Fig. 6a).

**Figure 7:**
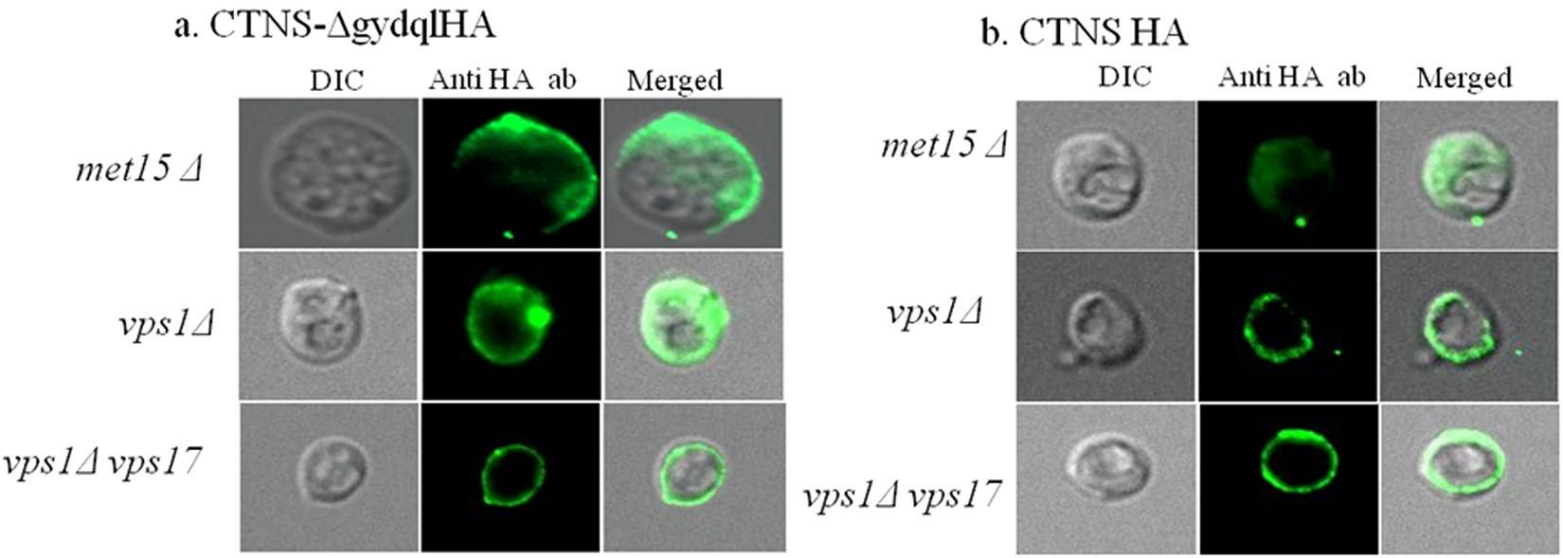
Cell surface localization studies of the CTNS and CTNS-Δgydql in different deletion backgrounds. Confocal analysis of *S*. *cerevisiae met15*Δ*yct1*Δ strain, *vps1*Δ and *vps1*Δ *vps17*Δ transformed with plasmid bearing HA epitope tagged *CTNS* or CTNS-∆gydql expressed under the TEF promoter. The transformants were fixed, permeabilized and labeled by indirect immunofluorescence using rabbit monoclonal anti-HA antibody and secondary antibody Alexa Flour^®^ 488 conjugated goat anti-mouse antibody (Molecular probes). Fluorescent images were obtained using a LEICA DM6000CS using a 64× oil objective.

We subsequently carried out a comparative radiolabelled uptake study of CTNS and CTNS-Δgydql in a *S*. *cerevisiae vps1*Δ strain versus *S*. *cerevisiae vps1*Δ*vps17*Δ and *S*. *cerevisiae vps1*Δ*ssh4*Δ backgrounds. Analysis of radiolabelled cystine uptake in the different backgrounds revealed maximal uptake in the *vps1*Δ*vps*17Δ background (Fig. 6b). *VPS17* which is a Subunit of the membrane-associated retromer complex is required for recruiting the retromer complex to the endosomal membranes^28^. Further analysis of C-terminally HA tagged CTNS and CTNS-Δgydql construct in comparison to the WT and *vps1*Δ deletion strains showed significantly localisation on the cell surface in *vps1*Δ*vps17*Δ mutant (Fig. 7). Surprisingly, in *S*. *cerevisiae vps1*Δ*vps17*Δ, CTNS WT also showed almost comparable uptake suggesting diversion of WT CTNS to plasma membrane to a significant extent (Fig. 7).

### Kinetics of WT and gain of function mutants

**Figure 8:**
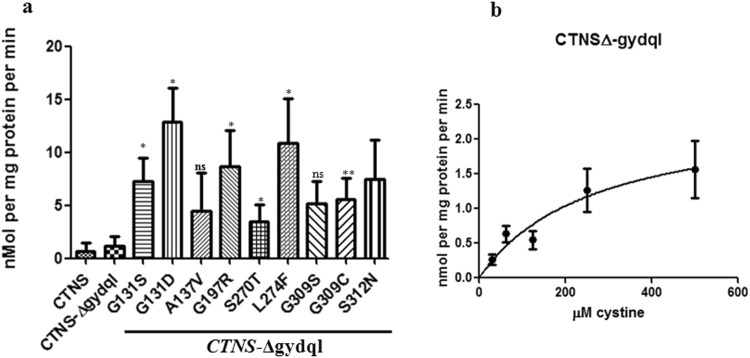
(**a**) Comparative uptake of gain of function mutants in *vps1*Δ*vps17*Δ background. The *vps1*Δ *vps17*Δ yeast strain (ABC 5017) was transformed with CTNS, *CTNS*-Δgydql and different gain-of-function mutants of *CTNS*-Δgydql and rate of uptake of cystine was measured 10 minutes and 20 time intervals in presence of 50 μM non-radioactive cystine. The cells were harvested at 10 and 20 min. intervals and results were plotted as nmol/mg protein/min after deducting the background uptake. The results represent the means of two different experiments, each in duplicate (±S.D., n = 4). Student’s T test was performed for the significance analysis. (**b**) Michaelis-Menten kinetics of *CTNS*-Δgydql constructs. The average *K*m (μM) and *V*max (nmol of cystine·mg·protein^−1^min^−1^) values were determined by non-linear regression analysis of *V* compared with [S] graphs showing saturation kinetics using GraphPad Prism Version 5.1 software. The experiment was repeated a minimum of two times for each test construct in duplicates for each cystine concentration (±S.D. n = 4).

Comparative analysis of the gain of function mutants by radiolabelled cystine uptake assay revealed an increased cystine uptake in all the gain of function mutants, confirming the observations seen with the growth assays on cystine medium (Fig. 8a).

We carried out a determination of the kinetic parameters using the *vps1*Δ*vps17*Δ background. The kinetic data in yeast showed significant variation from batch to batch. This could be due to accumulation of suppressors in the *vps1*Δ*vps17*Δ double deletion background since the secretory pathway is essential for the cell. We have subsequently standardized the conditions to use fresh transformants in the assay to obtain more consistent results. Analysis of kinetic parameters of CTNS-∆gydql in this background revealed a *K*m around 254.4 ± 61 µM (*V*max 2.7 ± 0.6 nmoles per min per mg protein) (n = 5) (Fig. 8b). In comparison, in mammalian cells, the *K*m was found to be around 278 ± 49 µM^9^ whereas in Xenopus oocyte studies the *K*m ranged between 75 µM (at pH 5.0) to about 225 µM (pH 6.5)^10^. In these cells, however, kinetic data is only available on the CTNS-Δgydql protein. This is owing to the difficulty in purifying lysosomes in mammals, kinetic data has not been obtained with the *CTNS* WT protein (containing the GYDQL domain). Since the CTNS WT protein could also confer some growth in the *vps1*Δ*vps17*Δ background, we therefore decided to evaluate this protein also. Although the uptake was lower, we did observe similar kinetic parameters (*K*m of 400 ± 133 µM).

We also subjected 5 mutants to kinetic analysis. These included the mutant G131S, G197R, S270T, L274F and G309C, all of which had showed significant increased functional activity when carrying out comparative uptakes (Figs 8a, S6).

We observed that for the mutants G131S and G309C the *K*m was 130.4 ± 10.9 µM (*V*max 34.2 ± 22.7 nmoles per min per mg protein) and 124.8 ± 23.5 µM (*V*max 4.9 ± 1.9 nmoles per min per mg protein) respectively. For gain of function mutant S270T, *K*m was found to be around 207.2 ± 41.5 µM (*V*max 33.5 ± 7.3 nmoles per min per mg protein), whereas for G197R it was found to be 97.3 ± 30.9 µM (*V*max 29.8 ± 13.2 nmoles per min per mg protein). Based on the kinetics we could observe a class of mutants with a significantly higher *V*max. However, it must be mentioned here that the increase in *V*max seen in this and a few other mutants although normalized to the total protein levels have not been corrected for variations in *CTNS* expression levels owing to the use of an untagged protein, and our inability to carry out westerns on these proteins.

### G197R and G309C mutants show a trafficking defect in *Saccharomyces cerevisiae*

The mutant G197R that was isolated in the gain-of-function mutant screen was also a mutant identified in patients with a milder form of ocular cystinosis. Thus, it was somewhat surprising that the mutant showed better uptake both in the comparative uptake as well as in the kinetic parameters. One explanation behind such observed discrepancy may be the different lipid environment or membrane potential of the plasma membranes from Xenopus oocyte (−40 to −60 mV) and *S*. *cerevisiae* cells (200 mV)^29^.

**Figure 9:**
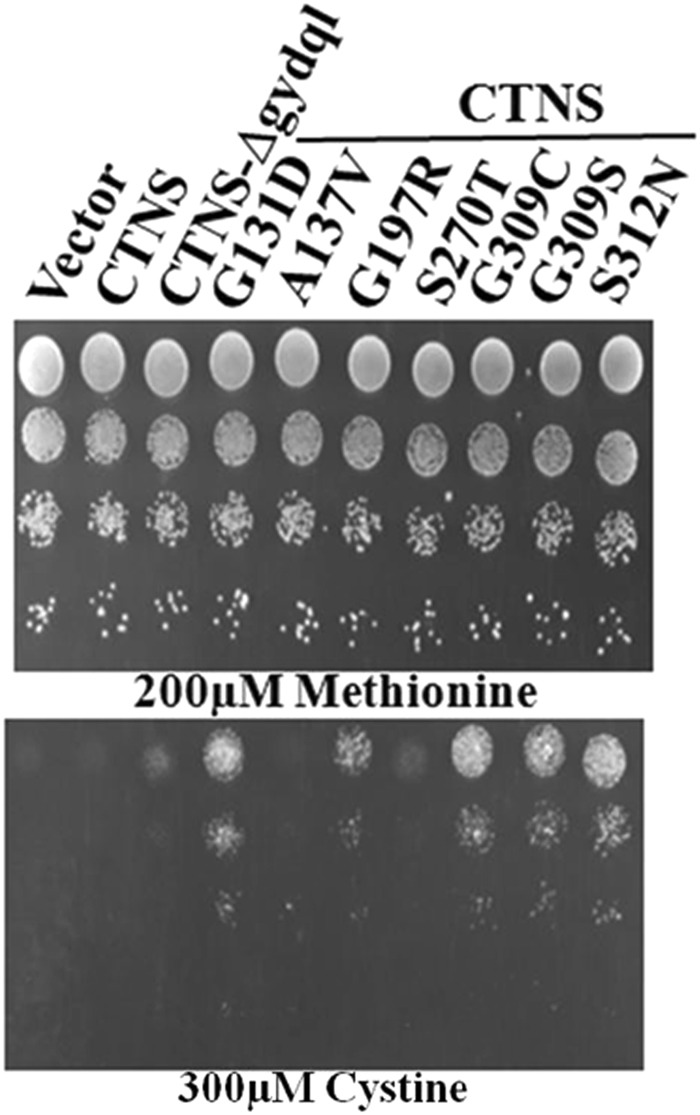
Functional analysis of reconstituted gain of function mutants in a CTNS WT background. The *met15*Δ*yct1*Δ yeast strain (ABC1580) was transformed with CTNS, CTNS-Δgydql and different mutants reconstituted in CTNS WT background were expressed under TEF promoter and examined by dilution spotting on minimal media containing 200 µM Methionine, & different concentration of cystine as described in experimental procedures. The photographs were taken after 2–3 days of incubation at 30 °C. The experiment was repeated with three independent transformations.

An alternate explanation could be that such mutants may not alter the functionality of the mutants, but affect its trafficking. We decided to evaluate this aspect using our assay. Since the mutations that were isolated in the genetic screen were isolated in a CTNS-Δgydql background, we decided to reintroduce these mutations in a *CTNS* background and examine if these mutants behaved like a CTNS-Δgydql protein, in being able to confer growth on cystine (Fig. 9). Gain of function mutants G131D, G197R, G309C/S and S312N were introduced into a CTNS gene containing the GYQDL region (WT CTNS). After reconstitution in the WT CTNS background, those mutants that are able to confer the ability to grow on cystine suggest their possible role in protein trafficking. We observed that G131D, G197R, G309C/S and S312N all enabled better growth even in a CTNS WT background. Further, the growth enhancement was even greater than that conferred by the deletion of the GYQDL motif. Only A137V did not confer growth, but this mutant also showed only marginal uptake in the radioactive assays. Particularly interesting was G197R, a patient mutation that was previously not known to have a trafficking defect.

## Discussion

In this study we describe the successful development of a yeast based assay for cystinosin function. We have exploited the inability of *S*. *cerevisiae met15*Δ strain to grow on cystine owing to the lack of a cystine transporter and have used this property as the basis for the growth based screen. Two aspects were critical for the success of such a screen. The first was that the human *CTNS* should be functional in yeast, and secondly it should be possible to mislocalize a functional *CTNS* transporter to the cell surface. A previous study has already reported that human *CTNS* was likely to be functional in yeast^30^. Thus the main requirement was to be able to mislocalize a functional protein to the cell surface. To address this, we used both a random mutagenesis approach (with the WT human *CTNS*), and a targeted mutagenesis approach (where we deleted targeting signals known for *CTNS* in mammalian cells). The interesting observation that emerged from both these approaches was that the intracellular retention of *CTNS* in yeast also depends on the GYQDL motif, and in fact on the precise residues, Y and L that are also crucial for the trafficking of this protein to the lysosome in human cells. This similarity of *CTNS* trafficking in both mammalian and yeast cells formed the basis for developing the yeast based growth assay for the isolation of mutants.

Indeed, despite the commonality in some of the trafficking features, evaluating the yeast assay for human cystinosin function by means of more quantitative kinetic parameters was crucial for subsequent analysis. Initial difficulties were encountered with the low uptake that was observed in the biochemical assays. The growth studies had earlier showed very distinct phenotypes between the human *CTNS* directed to the cell surface as compared to the control. However, these are done over several days, while uptake is done over a much shorter time scale (in minutes). In addition, incomplete cell surface localization of CTNS-Δgydql (a fact also observed in the mammalian cells) could also be a reason for the low uptake. Use of multicopy plasmids and constitutive stronger promoters (GPD) were also investigated to increase expression at the cell surfaced but as this led to a growth defect (data not shown) the approach had to be abandoned. Compounded with these issues was the low specific activity of ^14^C-cystine, and we eventually procured custom synthesized ^35^S-cystine of higher specific activity.

The use of the vacuolar biogenesis mutant backgrounds, the *vps1*Δ background alone and subsequently the use of the double mutant *vps* backgrounds was a breakthrough for these studies. Although this approach has not been attempted for other lysosomal transporters, we think that this could emerge as a general strategy to investigate some of these otherwise intractable lysosomal/vacuolar proteins. Using the *vps1*Δ*vps*17Δ background and the custom synthesized ^35^S cystine, the characterization of the *CTNS*, CTNS-Δgydql and the mutants could be carried out.

Despite reasonable uptake, reproducibility of the kinetic parameters was difficult to achieve. The reason for this variability could not be definitively established. One likely explanation is that the double mutants are not otherwise healthy and with time could be accumulating suppressors that would interfere with the reproducibility. Thus for consistent results, the plots generated with transformants which were less than a week old were considered for the analysis.

The WT CTNS-Δgydql in the yeast displayed a *K*m that was comparable to the reported *Km* for the CTNS-Δgydql in human cells^10^. Interestingly for the first time, we were also able to determine the kinetics of the full WT protein that contained even the GYQDL region and the *K*m was comparable to the value without the motif. This possibly represents the first report where the kinetics of the full protein has been described.

One of the major advantages of the yeast growth based assay is that it allows one to analyze existing mutants, and to isolate new mutants. We have used the screen to isolate mutants with the ability to grow faster on lower concentration of cystine. Functional characterization of these mutants would be expected to yield new insights on the *CTNS* protein.

Among the 9 different mutants that were obtained majority of the mutants were in the TMDs. Only one of the mutants, G197R was in the first luminal loop. Multiple sequence alignment with closer homologues revealed that G131, G197, S270, L274 and G309 are conserved from humans to *Drosophila melanogaster*. Comparison with clinical variants associated with cystinosis revealed that some of these residues such as S270 and G309 are clinically significant although the mutation isolated in this screen led to a slightly different change (S270Δ and G309D lead to infantile cystinosis). Interestingly, one of the mutants that we isolated in our screen was previously identified in patients. This was the G197R mutation that resulted in a mild form of ocular cystinosis in human patients^15^. This mutation was also the only one not on the transmembrane domain region, but is in the loop region facing inwardly within the vacuolar/lysosomal lumen. We observed that G197R showed a slight change in the *K*m, although it showed a much increased *V*max. G197R mutation is implicated in ocular cystinosis, and activity measurements had revealed that it had lower cystine transport ability than WT (but the activity was not null)^15^. The observations made here that the G197R mutation led to a significant trafficking defect, and was found to go to the cell surface even in the presence of the GYDQL motif, immediately raises the possibility that perhaps the reason for the mild cystinosis phenotype seen in patients bearing this mutation, is not the partial loss in activity, but the fact that they are mis-targeted and go to the plasma membrane.It would be interesting to see if these mutants behave similarly in the mammalian cell lines and Xenopus oocyte system in terms of mislocalization. G197R mutant has not been reported to be significantly mislocalized in the mammalian system, but considering it confers only a mild phenotype perhaps a more rigorous evaluation would be necessary. However, it is possible that some discrepancies may exist may be due to the differences in membrane environment or membrane potential of the both the systems^29^. The opening of these possibilities highlights the power of the yeast system to throw new light into these mutations and processes.

One limitation in this study has been the need to use the un-tagged protein since the C-terminal tag influenced the phenotype. However, very recently we have carried out comparisons of the tagged and un-tagged proteins of the CTNS-Δgydql protein in the *vps1*Δ *vps17*Δ background, and growth observations suggest that in the double delete background there was significant mislocalization to cell surface as seen by the growth studies in both the tagged and un-tagged constructs (Supplementary Fig. S7). If validated by the transport studies, this should be a further aid to the analysis of mutants by this yeast system.

A sequence homolog of *CTNS*, *ERS1* is present in *S*. *cerevisiae* (28% identical, 46% similarity), and has been shown to localize to the yeast vacuole. *S*. *cerevisiae ers1*Δ mutant has been reported to show hygromycin B (hygB) sensitivity which is suppressed by complementation of human *CTNS* gene but not by its non-functional mutant counterparts, thus highlighting the fact that, *ERS1* is a possible functional homolog of *CTNS*^30^. A recent report^31^ has shown that *ERS1*, functions as a yeast cystine transporter. However using the approaches and vacuolar mutant backgrounds described here we have not been able to confirm these findings. The exact reasons for these differences is not known at present (Shukla, Deshpande, Bachhawat, unpublished information).

Concluding, the yeast assay developed in this study will be useful for quickly deciphering the functionality of mutants of cystinosis. It would also be an excellent system for rapidly screening mutants where different kinds of screens can be set up. In the current study a specific screening strategy was employed that has enabled us to identify several residues that have a clear role in trafficking to the vacuole since mutations of these residues led to increased activity at the plasma membrane. In many of these mutants, the increased transport and significantly enhanced growth on cystine medium was far greater than what was observed when the GYQDL motif was deleted suggesting an important role for these residues. Previous studies had already suggested that additional motifs/residues are expected to be involved in targeting *CTNS* to the lysosome in mammalian cells, but these have been difficult to uncover. Thus, it would be valuable to follow up these initial insights that have been unearthed by this genetic screen. Further, the yeast screen with the possibility of doing suppressor analysis could provide insights into other domains and residues of *CTNS* and their functional significance.

## Experimental Procedures

### Chemicals and reagents-Chemicals and Reagents

All chemicals used were obtained from commercial sources and were of analytical grade. Media components, fine chemicals and reagents were purchased from Sigma Aldrich (St. Louis, USA), HiMedia (Mumbai, India), Merck Millipore India Ltd (Mumbai, India), USB Corporation (Ohio, USA) or Difco, USA. Cysteine and cystine were also purchased from Sigma Aldrich (St. Louis, USA). HA-Tag (6E2) Mouse monoclonal antibody and horse anti-mouse HRP-linked antibody were bought from Cell Signaling Technology (Danvers, MA, USA). Alexa Flour^®^ 488 conjugated goat anti-mouse antibody was obtained from Molecular probes (Eugene, Oregon, USA). Cysteine stock solutions that were prepared fresh were made by dissolving the required amount of cysteine in 1 ml of de-ionized water, which was then filter-sterilized using 0.2 μ filter membrane. Cystine stock solutions were prepared by dissolving the required amount of cystine in 1 ml of deionized water along with 25 µL of concentrated HCl, filter-sterilized using 0.2 μ filter membrane. ^14^C cystine and custom synthesized ^35^S cystine was procured from Perkin Elmer Ltd. (Boston, USA).

### Strains and Plasmids

*S*. *cerevisiae* strain MATα *ura3Δ0 leu2Δ0 his3Δ1 lys2Δ0 met15Δ::kanMX4* (ABC 733) was used as the parent host for all the transformations. This strain and most of the other *S*. *cerevisiae* strains used in this study are listed in Supplementary Table S1 were procured from Euroscarf. The yeast strains were maintained on yeast extract (1%), peptone (2%) and dextrose (2%) (YPD) medium and grown at 28–30 °C. The yeast transformants were selected and maintained on synthetic defined minimal medium containing yeast nitrogen base (0.17%), ammonium sulphate (0.5%) and dextrose (2%) supplemented with histidine, leucine, lysine, and uracil at 80 mg/l as per requirement. TEF-CTNS (ABE 3006) contained a WT CTNS downstream of the TEF–promoter in p416TEF vector. TEF-Δgydql (ABE 3513) contained a *CTNS* lacking C-terminal GYDQLN residues downstream of the TEF promoter in the centromeric vector p416TEF. These constructs and all other constructs generated in this study are listed in Supplementary Table S2.

#### Generation of vps1Δ deletion in different deletion backgrounds

Disruption of *vps1Δ* in different strains was carried out by means of a vps1Δ: HIS3 cassette where the his gene was amplified with flanking vps1 region using VPS1HIS3_F and VPS1HIS3_R primers. The amplified cassette was further transformed in the *S*. *cerevisiae* strains and the transformants were selected for the histidine prototrophy on SD media lacking histidine.

#### Construction of Site-directed mutants

The *CTNS*-Δgydql cloned downstream of the TEF promoter at *Bam*HI and *Xho*I site, of p416TEF vector (ABE 3513) was used as a template for generating site directed mutants and for which splice overlap extension strategy was utilized. The different mutagenic oligonucleotides pairs used for generation of these mutants are given in Supplementary Table S3.

### Random mutagenesis

Hydroxylamine based mutagenesis*-*The protocol used here was adopted from^32^. 10 μg plasmid DNA was dissolved in 0.5 ml of Hydroxylamine solution (90 mg NaOH, 350 mg hydroxylamine HCl in 5 ml water, pH around 6.5. freshly made up before use). This mixture was incubated at 37 °C for 20 hrs and the DNA was purified using Qiagen column. Finally the pool of mutagenized plasmid was directly transformed into the appropriate yeast strain.

### Growth Assays by dilution spotting

For growth assay, the different *S*. *cerevisiae* strains carrying the plasmid were grown overnight in minimal medium without uracil and re-inoculated in fresh medium to an OD_600_ of 0.1 and grown for 6 hours. The exponential phase cells were harvested washed with water and resuspended in water to an OD_600_ of 0.2. These were serially diluted to 1:10, 1:100 and 1:1000. 10 μl of these cell re-suspensions were spotted on minimal medium containing different concentrations of cysteine, cystine, methionine, as sole sulphur source. The plates were incubated at 30 °C for 3 to 4 days and photographs taken.

### Cellular Localization of HA-tagged CTNS

For localization studies, yeast cells were transformed with WT or mutant CTNS plasmid constructs under the TEF promoter. To localize CTNS and its different mutants indirect immunofluorescence was performed using a published protocol^33^. Transformants were grown to early exponential phase (OD_600_ ∼ 0.5) in minimal medium. 1 ml of the cell culture was harvested and fixed in 4% paraformaldehyde by 2 hours incubation at 30 °C. The fixed cells were washed twice with 100 mM potassium phosphate buffer (pH 6.5), and once with the same buffer containing 1.2 M sorbitol and finally re-suspended in 1 ml of this sorbitol buffer. Spheroplasting was done by addition of 10 µl β-mercaptoethanol and 20 µl 10X Lyticase (Sigma Aldrich) and incubation at 30 °C for 30 minutes. After washing the fixed spheroplasts with sorbitol buffer, they were re-suspended in the same buffer. 15 µl of the spheroplasts were adhered on to poly-lysine coated cover slips by incubation for 15 min at room temperature. The spheroplasts were permeabilized by treatment with 0.4% Triton X-100 in PBS, pH 7.3, for 2 min. This was followed by 30 min blocking in PBS buffer containing 1% BSA (w/v, USB, Cat# 70195) and overnight incubation with mouse monoclonal anti-HA primary antibody (Cat#2367 Cell Signaling Technology) at a dilution of 1:2,00 in the blocking buffer at 4 °C in a moist chamber. The primary antibody was removed and cover slips washed 10 times with the blocking buffer and incubated with secondary antibody Alexa Flour^®^ 488 conjugated goat anti-mouse antibody (Molecular probes) at 1:500 dilution for 4 hours at room temperature. Finally the cover slips were again washed 10 times with the blocking buffer and inverted onto a slide using VECTASHIELD (Vector labs, California, USA) antifade mounting medium. Fluorescent images were obtained using a LEICA DM6000CS using a 64× oil objective.

### Radioactive Cystine Transport assay for human lysosomal cystine transporter CTNS

*S*. *cerevisiae met15*Δ*yct1*Δ strain (ABC 1580) or *vps1*Δ (ABC 3740) or *vps1*Δ*vps17*Δ (ABC 5017) was transformed with WT pTEF-CTNS (ABE 3006), CTNS-Δgydql (ABE 3513) or the gain-of-function mutants, along with vector control p416TEF (ABE 433). Yeast transformants were grown overnight in minimal medium and re-inoculated to an OD_600_ of 0.1 in fresh minimal medium containing 0.2 mM methionine. Cultures were incubated at 30 °C for 6–8 hours and exponentially growing cells were harvested and washed with a large volume of ice-cold MES buffer (pH 5.5). Cells were finally re-suspended in the MES buffer containing 2% glucose (resuspension buffer) at 3. 5 OD_600_/ml, aliquoted in 240 μl samples and kept on ice. After 5-minute pre-incubation of cells at 30 °C, the uptake was initiated by addition of 240 μl of assay medium, which contained the radiolabelled cystine. The assay medium contained radiolabelled cystine (^35^S-cystine, specific activity2100 Ci mmol^−1^) in MES re-suspension buffer (pH 5.5), at the final concentration of cystine was 50 µM in the reaction vial. At 10 minutes and 20 minutes time points (up to which the uptake was linear), the uptake was stopped by diluting the medium with a 20-fold volume of ice-cold water and cells were collected on the glass fibre pre filter by vacuum filtration. The harvested cells were washed twice with the same volume of ice-cold water. The filters were immersed in 3 ml of scintillation fluid and radioactivity was measured using liquid scintillation counter. The results were expressed as nmoles of cystine·mg.protein^−1^ min^−1^. For the measurements of protein the 100 μl of the above cell suspension (cell suspension volume used for the transport assay) was boiled with sodium hydroxide and total protein estimated by using the Bradford reagent (Sigma Aldrich) with bovine serum albumin as a standard. The transport activity for the different gain-of-function mutants of *CTNS* was expressed as percentage activity relative to the wild-type protein. Student’s T test was performed for the significance analysis. For saturation kinetics of gain-of-function mutants were transformed in *S*. *cerevisiae vps1Δvps17* (ABC 5017) along with the WT-CTNS-Δgydql and p416TEF vector control. For the analysis of saturation kinetics, ^35^S cystine of specific activity 2100 Ci mmol^−1^ was used. The initial rate of cystine uptake was measured at a range of cystine concentrations from 31.25 µM to 500 µM, with specific activity being kept constant at each concentration. The initial rate of cystine uptake was determined by measuring the radioactive cystine accumulated in the cells at 20 minutes and 40 minutes time points. After subtracting the background uptake observed in the vector control at the similar time points, rate of cystine uptake corresponding to *CTNS* and its mutants was calculated and used to plot Michaelis-Menten plot for determining the kinetic parameters.

## Electronic supplementary material

Dataset 1
