# Author Correction: A Genetic Screen for Investigating the Human Lysosomal CystineTransporter, Cystinosin

**DOI:** 10.1038/s41598-019-50845-2

**Published:** 2019-10-15

**Authors:** Anup Arunrao Deshpande, Anuj Shukla, Anand Kumar Bachhawat

**Affiliations:** Indian Institute of Science and Education Research Mohali, Sector 81, Knowledge City, SAS Nagar, Punjab India

Correction to: *Scientific Reports* 10.1038/s41598-018-21483-x, published online 21 February 2018

This Article contained errors.

As a result of a figure assembly error, in Figure 5b subpanel showing samples vps1Δ through vaps8Δ was duplicated for vps25Δ through vps31Δ and vps53Δ through vps63Δ, for methionine treated samples. This same methionine sample was correctly shown in the Supplementary Information File.

Additionally, in the Supplementary Information file, as a result of labelling error, the label for vps32Δ through vps38Δ was duplicated for subsequent subpanel which instead should be labelled as vps39Δ through vps52Δ.

These errors have now been corrected in the PDF and HTML versions of the Article, and in the accompanying Supplementary Information File.

