# Supplementary material for: A Genetic Screen for Investigating the Human Lysosomal CystineTransporter, Cystinosin
Source: Sci Rep. 2018 Feb 21;8:3442. doi: 10.1038/s41598-018-21483-x (PMC5821828; doi:10.1038/s41598-018-21483-x)
Supplement: Supplementary file 1 — Dataset 1 [file 41598_2018_21483_MOESM1_ESM.docx]

**A Genetic Screen for Investigating the Human Lysosomal Cystine Transporter, Cystinosin**

Anup A Deshpande^1^, Anuj Shukla^1^ and Anand K Bachhawat*

**Supplementary Information**

**Table S1: list of Strains used in this study**

| Strain | Genotype | | Source |
| --- | --- | --- | --- |
| ***Escherichia coli strain*** | | | |
| ABE 460 (DH5α) | *F^-^ gyrA96*(Nal) *recA1 relA1 endA1 thi-1 hsdR17* (r_k_^-^m_k_^+^) *glnV44 deoR*Δ *(lacZYA-argF) U169* [φ80dΔ *(lacZ) M15*] | | Lab strain |
| ***Saccharomyces cerevisiae* Strains** | | | |
| ABC 733 (BY4741) | | *MATa his3Δ1 leu2Δ0 met15Δ ura3Δ0* | J. Boeke |
| ABC 3740 (*vps1Δ)* | | *MATa his3Δ1 leu2Δ0 met15Δ ura3Δ0 YKR001c:: kanMX4* | Euroscarf |
| ABC4160 (*vps8Δ*) | | *MATa his3Δ1 leu2Δ0 met15Δ ura3Δ0 YAL002w:: kanMX4* | Euroscarf |
| ABC4161 (*vps11Δ*) | | *MATa his3Δ1 leu2Δ0 met15Δ ura3Δ0 YMR231w:: kanMX4* | Euroscarf |
| ABC4162 (*vps29Δ*) | | *MATa his3Δ1 leu2Δ0 met15Δ ura3Δ0 YHR012w:: kanMX4* | Euroscarf |
| ABC4163 (*vps75Δ*) | | *MATa his3Δ1 leu2Δ0 met15Δ ura3Δ0 YNL246w:: kanMX4* | Euroscarf |
| ABC4164 (*vps35Δ*) | | *MATa his3Δ1 leu2Δ0 met15Δ ura3Δ0 YJL154c::kanMX4* | Euroscarf |
| ABC4165 (*vps26Δ*) | | *MATa his3Δ1 leu2Δ0 met15Δ ura3Δ0 YJL053w:: kanMX4* | Euroscarf |
| ABC4166 (*vps13Δ*) | | *MATa his3Δ1 leu2Δ0 met15Δ ura3Δ0 YLL040c:: kanMX4* | Euroscarf |
| ABC4167 (*vps32Δ*) | | *MATa his3Δ1 leu2Δ0 met15Δ ura3Δ0 YLR025w:: kanMX4* | Euroscarf |
| ABC4168 (*vps6Δ*) | | *MATa his3Δ1 leu2Δ0 met15Δ ura3Δ0 YOR036w:: kanMX4* | Euroscarf |
| ABC4169 (*vps21Δ*) | | *MATa his3Δ1 leu2Δ0 met15Δ ura3Δ0 YOR089c:: kanMX4* | Euroscarf |
| ABC4170 (*vps30Δ*) | | *MATa his3Δ1 leu2Δ0 met15Δ ura3Δ0 YPL120w:: kanMX4* | Euroscarf |
| ABC4171 (*vps17Δ*) | | *MATa his3Δ1 leu2Δ0 met15Δ ura3Δ0 YOR132W:: kanMX4* | Euroscarf |
| ABC4172 (*vps25Δ*) | | *MATa his3Δ1 leu2Δ0 met15Δ ura3Δ0 YJR102C:: kanMX4* | Euroscarf |
| ABC4173 (*vps37Δ*) | | *MATa his3Δ1 leu2Δ0 met15Δ ura3Δ0 YLR119w:: kanMX4* | Euroscarf |
| ABC4174 (*vps28Δ*) | | *MATa his3Δ1 leu2Δ0 met15Δ ura3Δ0 YPL065w:: kanMX4* | Euroscarf |
| ABC4175 (*vps16Δ*) | | *MATa his3Δ1 leu2Δ0 met15Δ ura3Δ0 YPL045w:: kanMX4* | Euroscarf |
| ABC4176 (*vps22Δ*) | | *MATa his3Δ1 leu2Δ0 met15Δ ura3Δ0 YPL002C:: kanMX4* | Euroscarf |
| ABC4177 (*vps10Δ*) | | *MATa his3Δ1 leu2Δ0 met15Δ ura3Δ0 YBL017c:: kanMX4* | Euroscarf |
| ABC4178 (*vps23Δ*) | | *MATa his3Δ1 leu2Δ0 met15Δ ura3Δ0 YCL008c:: kanMX4* | Euroscarf |
| ABC4179 (*vps39Δ*) | | *MATa his3Δ1 leu2Δ0 met15Δ ura3Δ0 YDL077c:: kanMX4* | Euroscarf |
| ABC4180 (*vps54Δ*) | | *MATa his3Δ1 leu2Δ0 met15Δ ura3Δ0 YDR027c:: kanMX4* | Euroscarf |
| ABC4181 (*vps61Δ*) | | *MATa his3Δ1 leu2Δ0 met15Δ ura3Δ0 YDR136c:: kanMX4* | Euroscarf |
| ABC4182 (*vps18Δ*) | | *MATa his3Δ1 leu2Δ0 met15Δ ura3Δ0 YLR148w: :kanMX4* | Euroscarf |
| ABC4183 (*vps44Δ*) | | *MATa his3Δ1 leu2Δ0 met15Δ ura3Δ0 YDR456w:: kanMX4* | Euroscarf |
| ABC4184 (*vps52/67Δ*) | | *MATa his3Δ1 leu2Δ0 met15Δ ura3Δ0 YDR484w:: kanMX4* | Euroscarf |
| ABC4185 (*vps45Δ*) | | *MATa his3Δ1 leu2Δ0 met15Δ ura3Δ0 YGL095c:: kanMX4* | Euroscarf |
| ABC4186 (*vps73Δ*) | | *MATa his3Δ1 leu2Δ0 met15Δ ura3Δ0 YLR148w:: kanMX4* | Euroscarf |
| ABC4187 (*vps*43*Δ*) | | *MATa his3Δ1 leu2Δ0 met15Δ ura3Δ0 YGL212w:: kanMX4* | Euroscarf |
| ABC4188 (*vps62Δ*) | | *MATa his3Δ1 leu2Δ0 met15Δ ura3Δ0 YGR141w:: kanMX4* | Euroscarf |
| ABC4189 (*vps24Δ*) | | *MATa his3Δ1 leu2Δ0 met15Δ ura3Δ0 YKL041w:: kanMX4* | Euroscarf |
| ABC4190 (*vps51Δ*) | | *MATa his3Δ1 leu2Δ0 met15Δ ura3Δ0 YKR020w:: kanMX4* | Euroscarf |
| ABC4191 (*vps34Δ*) | | *MATa his3Δ1 leu2Δ0 met15Δ ura3Δ0 YLR240w:: kanMX4* | Euroscarf |
| ABC4192 (*vps63Δ*) | | *MATa his3Δ1 leu2Δ0 met15Δ ura3Δ0 YLR261w:: kanMX4* | Euroscarf |
| ABC4193 (*vps65Δ*) | | *MATa his3Δ1 leu2Δ0 met15Δ ura3Δ0 YLR322w:: kanMX4* | Euroscarf |
| ABC4194 (*vps38Δ*) | | *MATa his3Δ1 leu2Δ0 met15Δ ura3Δ0 YLR360w:: kanMX4* | Euroscarf |
| ABC4195 (*vps36Δ*) | | *MATa his3Δ1 leu2Δ0 met15Δ ura3Δ0 YLR417w:: kanMX4* | Euroscarf |
| ABC4196 (*vps27Δ*) | | *MATa his3Δ1 leu2Δ0 met15Δ ura3Δ0 YNR006w:: kanMX4* | Euroscarf |
| ABC4197 (*vps69Δ*) | | *MATa his3Δ1 leu2Δ0 met15Δ ura3Δ0 YPR087w:: kanMX4* | Euroscarf |
| ABC4198 (*vps66Δ*) | | *MATa his3Δ1 leu2Δ0 met15Δ ura3Δ0 YPR139c:: kanMX4* | Euroscarf |
| ABC4199 (*vps20Δ*) | | *MATa his3Δ1 leu2Δ0 met15Δ ura3Δ0 YMR077c:: kanMX4* | Euroscarf |
| ABC4200 (*vps68Δ*) | | *MATa his3Δ1 leu2Δ0 met15Δ ura3Δ0 YOL129w:: kanMX4* | Euroscarf |
| ABC4201 (*vps9Δ*) | | *MATa his3Δ1 leu2Δ0 met15Δ ura3Δ0 YML097c:: kanMX4* | Euroscarf |
| ABC4202 (*vps71Δ*) | | *MATa his3Δ1 leu2Δ0 met15Δ ura3Δ0 YML041c:: kanMX4* | Euroscarf |
| ABC4203 (*vps53Δ*) | | *MATa his3Δ1 leu2Δ0 met15Δ ura3Δ0 YJL029c::kanMX4* | Euroscarf |
| ABC4204 (*vps55Δ*) | | *MATa his3Δ1 leu2Δ0 met15Δ ura3Δ0 YJR044c:: kanMX4* | Euroscarf |
| ABC4205 (*vps70Δ*) | | *MATa his3Δ1 leu2Δ0 met15Δ ura3Δ0 YJR126c:: kanMX4* | Euroscarf |
| ABC4206 (*vps5Δ*) | | *MATa his3Δ1 leu2Δ0 met15Δ ura3Δ0 YOR069w:: kanMX4* | Euroscarf |
| ABC4207 (*vps72Δ*) | | *MATa his3Δ1 leu2Δ0 met15Δ ura3Δ0 YDR485c:: kanMX4* | Euroscarf |
| ABC4072 (*vps*2*Δ*) | | *MATa his3Δ1 leu2Δ0 met15Δ ura3Δ0 YKL002w:: kanMX4* | Euroscarf |
| ABC4073 (*vps46Δ*) | | *MATa his3Δ1 leu2Δ0 met15Δ ura3Δ0 YKR035w-A:: kanMX4* | Euroscarf |
| ABC4074 (*vps4Δ*) | | *MATa his3Δ1 leu2Δ0 met15Δ ura3Δ0 YPR173-c:: kanMX4* | Euroscarf |
| ABC4075 (*vps33Δ*) | | *MATa his3Δ1 leu2Δ0 met15Δ ura3Δ0 YLR396-c:: kanMX4* | Euroscarf |
| ABC4076 (*vps15Δ*) | | *MATa his3Δ1 leu2Δ0 met15Δ ura3Δ0 YBR097-w:: kanMX4* | Euroscarf |
| ABC4077 (*vps64Δ*) | | *MATa his3Δ1 leu2Δ0 met15Δ ura3Δ0 YPR173-c:: kanMX4* | Euroscarf |
| ABC4078 (*vps19Δ*) | | *MATa his3Δ1 leu2Δ0 met15Δ ura3Δ0 YDR323-c:: kanMX4* | Euroscarf |
| ABC4079 (*vps74Δ*) | | *MATa his3Δ1 leu2Δ0 met15Δ ura3Δ0 YDR372-c:: kanMX4* | Euroscarf |
| ABC4080 (*vps60Δ*) | | *MATa his3Δ1 leu2Δ0 met15Δ ura3Δ0 YDR486-c:: kanMX4* | Euroscarf |
| ABC4081 (*vps3Δ*) | | *MATa his3Δ1 leu2Δ0 met15Δ ura3Δ0 YDR495-c:: kanMX4* | Euroscarf |
| ABC4220 (*vps31Δ*) | | *MATa his3Δ1 leu2Δ0 met15Δ ura3Δ0 YPL084-w:: kanMX4* | Euroscarf |
| ABC4221 (*vps41Δ*) | | *MATa his3Δ1 leu2Δ0 met15Δ ura3Δ0 YDR080-w:: kanMX4* | Euroscarf |
| ABC5016 (*vps1Δ ssh4Δ*) | | *MATa leu2Δ0 met15Δ ura3Δ0 YKL124W::kanMX4 YKR001c::HIS3* | This study |
| ABC5017(*vps1Δ vps17Δ*) | | *MATa leu2Δ0 met15Δ ura3Δ0 YOR132W::kanMX4 YKR001c::HIS3* | This study |
| ABC5018(*vps1Δ pep1Δ*) | | *MATa leu2Δ0 met15Δ ura3Δ0 YBL017C::kanMX4 YKR001c::HIS3* | This study |
| ABC5019(*vps1Δ vps27Δ*) | | *MATa leu2Δ0 met15Δ ura3Δ0 YNR006::kanMX4 YKR001c::HIS3* | This study |
| ABC5020(*vps1Δ clc1Δ*) | | *MATa leu2Δ0 met15Δ ura3Δ0::kanMX4 YKR001c::HIS3* | This study |
| ABC5021(*vps1Δ erd1Δ*) | | *MATa leu2Δ0 met15Δ ura3Δ0::kanMX4 YKR001c::HIS3* | This study |
| ABC5022 (*vps1Δ doa4Δ*) | | *MATa leu2Δ0 met15Δ ura3Δ0YDR069W::kanMX4 YKR001c::HIS3* | This study |
| ABC5023 (*vps1Δ vps44Δ* | | *MATa leu2Δ0 met15Δ ura3Δ0 YDR453W::kanMX4 YKR001c::HIS3* | This study |

**Table S2: List of plasmids used in this study**

| **Strain** | **Genotype** | **Source** |
| --- | --- | --- |
| **p416TEF** | ABE 443 | The CEN-vector bearing *URA3* marker and TEF promoter-MCS-terminator for yeast expression and Amp^r^ marker for selection in *E. coli* ([Mumberg et al., 1995](#_ENREF_20)). |
| **p416TEF-*CgCYN1*HA** | ABE 2847 | The plasmid bearing C-terminus HA tagged *CgCYN1* at *Bam*HI and *Xho*I sites of p416TEF (ABE 443). (Clone constructed previously in lab) |
| ***CTNS*WT** | ABE3006 | Human lysosomal cystine transporter *CTNS* CDNA cloned at at *Bam*HI and *Xho*I sites of p416TEF (ABE 443). (Clone constructed previously in lab) |
| ***CTNS* Δ-GYDQL** | ABE3513 | Human lysosomal cystine transporter *CTNS* lacking C-terminal lysosomal targeting motif GYDQL(N) cloned at at*Bam*HI and *Xho*I sites of p416TEF (ABE 443) |
| ***CTNS*HA** | ABE3480 | Human lysosomal cystine transporter *CTNS* with C-terminal HA tag cloned at *Bam*HI and *Xho*I sites of p416TEF (ABE 443) |
| ***CTNS*Δ-GYDQLHA** | ABE3481 | *CTNS* Δ-GYDQL (ABE3513) with C-terminal HA tag cloned at *Bam*HI and *Xho*I sites of p416TEF (ABE 443) |
| ***CTNS*Δ-YFPQAHA** | ABE3485 | *CTNS*HA (ABE3480) lacking YFPQA lysosomal motif cloned at *Bam*HI and *Xho*I sites of p416TEF (ABE 443) |
| ***CTNS*-ΔGYDQLΔYFPQAHA** | ABE3486 | *CTNS* Δ-GYDQLHA (ABE3481) lacking YFPQA lysosomal motif cloned at *Bam*HI and *Xho*I sites of p416TEF (ABE 443) |
| ***CTNS*Q365Δ** | ABE3487 | *CTNS*WT (ABE3006) hydroxylamine mutant with Q365 converted to nonsense codon. |
| ***CTNS*5thHAgydqlΔ** | ABE3514 | *CTNS* Δ-GYDQL (ABE3513) with HA tag in the 5^th^ loop (cytosolic loop) |
| ***CTNS*5thHAWT** | ABE3515 | *CTNS*WT (ABE3006) with HA tag in the 5^th^ loop (cytosolic loop) |
| **G309C** | ABE3516 | *CTNS* Δ-GYDQL(ABE3513) with G309C gain-of-function mutation |
| **G131S** | ABE4851 | *CTNS* Δ-GYDQL (ABE3513) with G131S gain-of-function mutation |
| **G131D** | ABE4852 | *CTNS* Δ-GYDQL(ABE3513) with G131D gain-of-function mutation |
| **G197R** | ABE4853 | *CTNS* Δ-GYDQL(ABE3513) with G197R gain-of-function mutation |
| **S270T** | ABE4854 | *CTNS* Δ-GYDQL(ABE3513) with S270T gain-of-function mutation |
| **L274F** | ABE4855 | *CTNS* Δ-GYDQL(ABE3513) with L274F gain-of-function mutation |
| **G309S** | ABE4856 | *CTNS* Δ-GYDQL(ABE3513) with G309S gain-of-function mutation |
| **G131A** | ABE4858 | *CTNS* Δ-GYDQL(ABE3513)with G131A mutation |
| **G197A** | ABE4859 | *CTNS* Δ-GYDQL(ABE3513) with G197A mutation |
| **S270A** | ABE4860 | *CTNS* Δ-GYDQL(ABE3513) with S270A mutation |
| **L274A** | ABE4861 | *CTNS* Δ-GYDQL(ABE3513) with L274A mutation |
| **G309A** | ABE4862 | *CTNS* Δ-GYDQL(ABE3513) with G309A mutation |
| ***CTNS*_S141F** | ABE4865 | *CTNS* Δ-GYDQL(ABE3513) with S141F mutation |

**Table S3: List of Primers used in this study**

| **Primer name** | **Sequence (5’-3’)** |
| --- | --- |
| p416TEF_F | TTGATATTTAAGTTAATAAACGG |
| p416TEF_R | TTCAGGTTGTCTAACTCCTTC |
| H_CTNS_F | CTAAGAGGATCCATGATAAGGAATTGGCTGAC |
| H_CTNS_R | CTAAGACTCGAGCTAGTTCAGCTGGTCATAC |
| CTNSgydqlΔ_R | TCGACACTCGAGCTACGGTCTCTTTCTGTACAAAC |
| CTNS_YFPQA_F | CTCGCAGTCACGCTGGTCAAGTACATGAACTTTTACTACAAAAGCAC |
| CTNS_YFPQA_R | CAGTGCTTTTGTAGTAAAAGTTCATGTACTTGACCAGCGTGACTGCG |
| HCTNSHA_R | ATATAAACTCGAGCTATGCATAATCAGGAACATCATATGGATAGTTCAGCTGGTCATAC |
| HCTNSDELHA_R | ATATAACTCGAGCTATGCATAATCAGGAACATCATATGGATACGGTCTCTTTCTGTAC |
| CTNS_5th HAF | TTCCACAGGCCTACATGAACTATCCATATGATGTTCCTGATTATGCATTTTACTACAAAAGCACTG |
| CTNS_5 th HAR | CTCAGTGCTTTTGTAGTAAAATGCATAATCAGGAACATCATATGGATAGTTCATGTAGGCCTGTGG |
| G131S_F | ATCATAAACCAGGTGATTAGCTGGATCTACTTTGTGGCCTGGTCC |
| G131S_R | GATGGACCAGGCCACAAAGTAGATCCAGCTAATCACCTGGTTTATG |
| G131D_F | ATCATAAACCAGGTGATTGACTGGATCTACTTTGTGGCCTGGTCC |
| G131D_R | ACCAGGCCACAAAGTAGATCCAGTCAATCACCTGGTTTATGATGC |
| G197R_F | CTCCTCAAATACCCCAACAGAGTGAACCCCGTGAACAGCAACG |
| G197R_R | TTGCTGTTCACGGGGTTCACTCTGTTGGGGTATTTGAGGAGAAAC |
| S270T_F | GCAGTTTCTCTTCTGCTTCACCTACATCAAGCTCGCAGTCAC |
| S270T_R | GACTGCGAGCTTGATGTAGGTGAAGCAGAAGAGAAACTGCAG |
| L274F_F | CTGCTTCTCCTACATCAAGTTCGCAGTCACGCTGGTCAAGTATTTTC |
| L274F_R | AATACTTGACCAGCGTGACTGCGAACTTGATGTAGGAGAAGCAGAAG |
| G309S_F | CTCCTGGACTTCACCGGGAGCAGCTTCAGCCTCCTGCAGATG |
| G309S_R | TCTGCAGGAGGCTGAAGCTGCTCCCGGTGAAGTCCAGGAGCAC |
| G309C_F | CTCCTGGACTTCACCGGGTGCAGCTTCAGCCTCCTGCAGATG |
| G309C_R | TCTGCAGGAGGCTGAAGCTGCACCCGGTGAAGTCCAGGAGCAC |
| CTNS_ V89A_F | GACAAACTCCTCTTTTCAAGCGACATCTCAAAATGTTGGACAAC |
| CTNS _V89A_R | TGTCCAACATTTTGAGATGTCGCTTGAAAAGAGGAGTTTGTCAC |
| CTNS_G131A_F | ATCATAAACCAGGTGATTGCCTGGATCTACTTTGTGGCCTGGTCC |
| CTNS_G131A_R | GATGGACCAGGCCACAAAGTAGATCCAGGCAATCACCTGGTTTATG |
| CTNS_G197A_F | CTCCTCAAATACCCCAACGCAGTGAACCCCGTGAACAGCAACG |
| CTNS_G197A_R | TTGCTGTTCACGGGGTTCACTGCGTTGGGGTATTTGAGGAGAAAC |
| CTNS_L274A_F | CTGCTTCTCCTACATCAAGGCCGCAGTCACGCTGGTCAAGTATTTTC |
| CTNS_L274A_R | AATACTTGACCAGCGTGACTGCGGCCTTGATGTAGGAGAAGCAGAAG |
| CTNS_S270A_F | GCAGTTTCTCTTCTGCTTCGCCTACATCAAGCTCGCAGTCAC |
| CTNS_S270A_R | GACTGCGAGCTTGATGTAGGCGAAGCAGAAGAGAAACTGCAG |
| CTNS_G309A_F | CTCCTGGACTTCACCGGGGCCAGCTTCAGCCTCCTGCAGATG |
| CTNS_G309A_R | TCTGCAGGAGGCTGAAGCTGGCCCCGGTGAAGTCCAGGAGCAC |
| CTNS S141F_F | TTTGTGGCCTGGTCCATCTTCTTCTACCCTCAGGTGATCATGAATTG |
| CTNS S141F_R | TCATGATCACCTGAGGGTAGAAGAAGATGGACCAGGCCACAAAGTAG |

**Table S4: Gain- of-function mutants of CTNS: Frequency of isolation, location and conservation**

| AA Change | No of times (in WT ) | No of times (in vps ) | Conservation | Location | Region |
| --- | --- | --- | --- | --- | --- |
| G131D | 1 | 1 | *Hs, Mm, Xl, Dr,Dm* | TM1 | PQ loop1 |
| G131S | 1 | 0 | *Hs, Mm, Xl Dr, Dm* | TM1 | PQ loop1 |
| A137V | 0 | 1 | *Hs, Mm, Xl, Dr,Dm* | TM1 | PQ loop1 |
| G197R | 1 | 3 | *Mm, Xl,Dr, Dm,* | 2^nd^interloop |  |
| S270T | 1 | 0 | *Hs, Mm, Xl, Dm* | TM5 | PQ loop2 |
| L274F | 4 | 3 | *Hs, Mm, Dr,* | TM5 | PQ loop2 |
| G309 C | 2 | 0 | *Hs, Mm, Xl, Dr, Dm* | TM6 | PQ loop2 |
| G309 S | 2 | 3 | *Hs, Mm, Xl, Dr, Dm* | TM6 | PQ loop2 |
| S312N | 0 | 2 | *Hs, Mm, Xl, Dr, Dm* | TM6 | PQ loop2 |

(*Hs*:*H. sapiens*, *Mm*: *M. musculus*, *Xl*: *X. laevis*, *Dr*: *D. rerio, Dm*: *D. melanogaster,* )


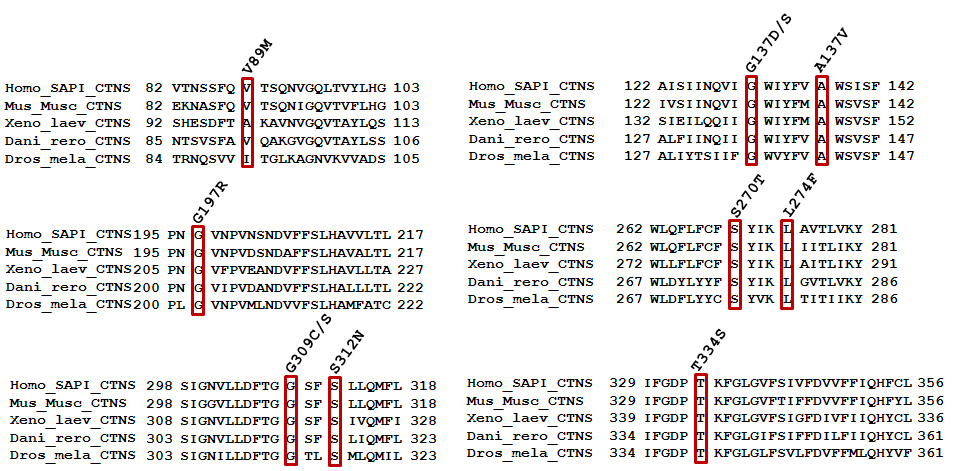


**Fig.S1 Multiple sequence alignment for determining the conservation pattern for the gain of function mutants**

Gain-of- function mutants identified through the mutagenesis screen have been clubbed by a bracket.

Multiple sequence alignment of the TMDs of lysosomal cystine transporters including, *H. sapiens* (*Homo_ sapi*_CTNS: NP_004928), *M. musculus* (*Mus_musc*_CTNS: NP_112541.1), *X. laevis* (Xeno_laev_CTNS:NP_001084833.1), *D. rario ( Dani_rero*_CTNS:NP_001018407.1), *D. melanogaster* (Dros_mela_CTNS NP_651116.1) using ClustalW. Conservation of gain of function mutants identified through screen are bracketed.


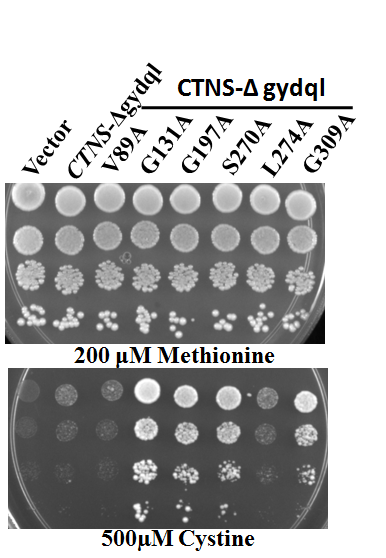


**Fig.S2 Functional analysis of alanine mutants of gain-of-function mutants**

The *met15Δyct1Δ* yeast strain (ABC 1580) was transformed with, *CTNS*-Δgydql and alanine mutants of residues picked up as gain-of-function mutants under the TEF promoter, along with the control vector and examined by dilution spotting on minimal media containing 200 µM Methionine, and 500 µM L-cystine.V89, which was initially isolated as a mutant but its phenotype could not be confirmed, and is included in this analysis.


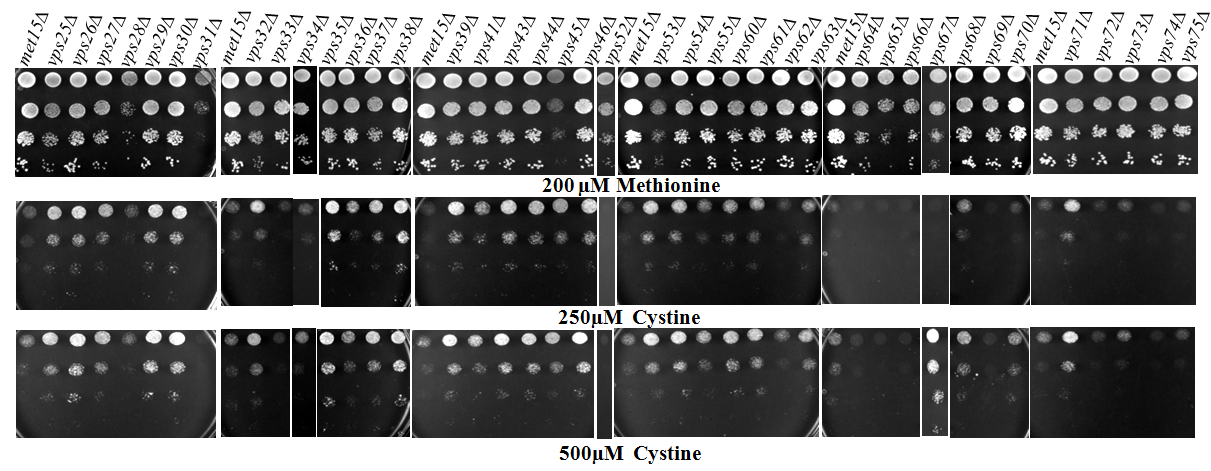


**Fig. S3 Functional analysis of TEF-CTNS-Δgydql transformed in different vacuolar protein sorting defective mutants on medium containing different concentration of cystine.**

Different vacuolar protein sorting defective mutants were transformed with, CTNS-Δgydql expressed under the TEF promoter, and examined by dilution spotting on minimal media containing 200µM Methionine (control plates) and different concentrations of cystine.


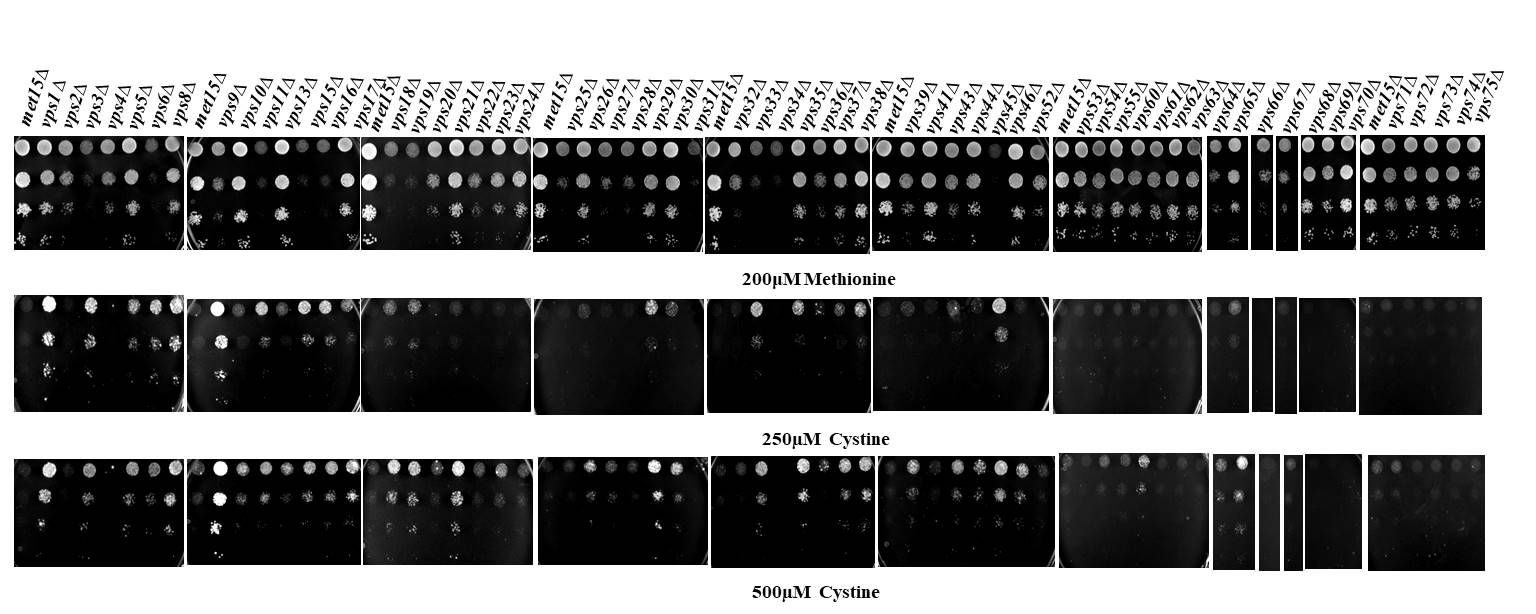


**Fig. S4 Functional analysis of TEF-CTNS construct transformed in different vacuolar protein sorting defective mutants on medium containing different concentration of cystine.**

Different vacuolar protein sorting defective mutants were transformed with, CTNS expressed under the TEF promoter, and examined by dilution spotting on minimal media containing 200µM Methionine (control plates), and different concentrations of cystine.

**
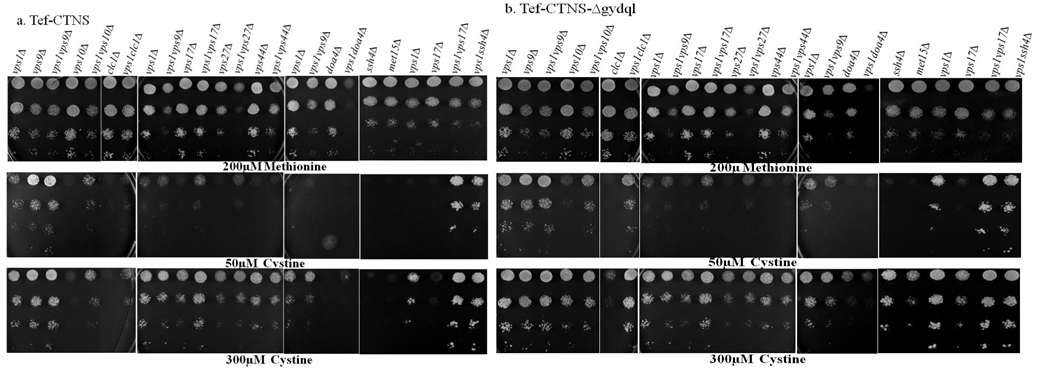
**

**Fig. S5 Functional analysis of a) TEF-CTNS and b)TEF-CTNS -Δgydql construct transformed in different vacuolar protein sorting defective mutants (double delete) on medium containing different concentration of cystine.**

Different vacuolar protein sorting defective mutants transformed with, CTNS expressed under TEF promoter, and examined by dilution spotting on minimal media containing 200µM Methionine, & different concentration of cystine as described in experimental procedures.

**
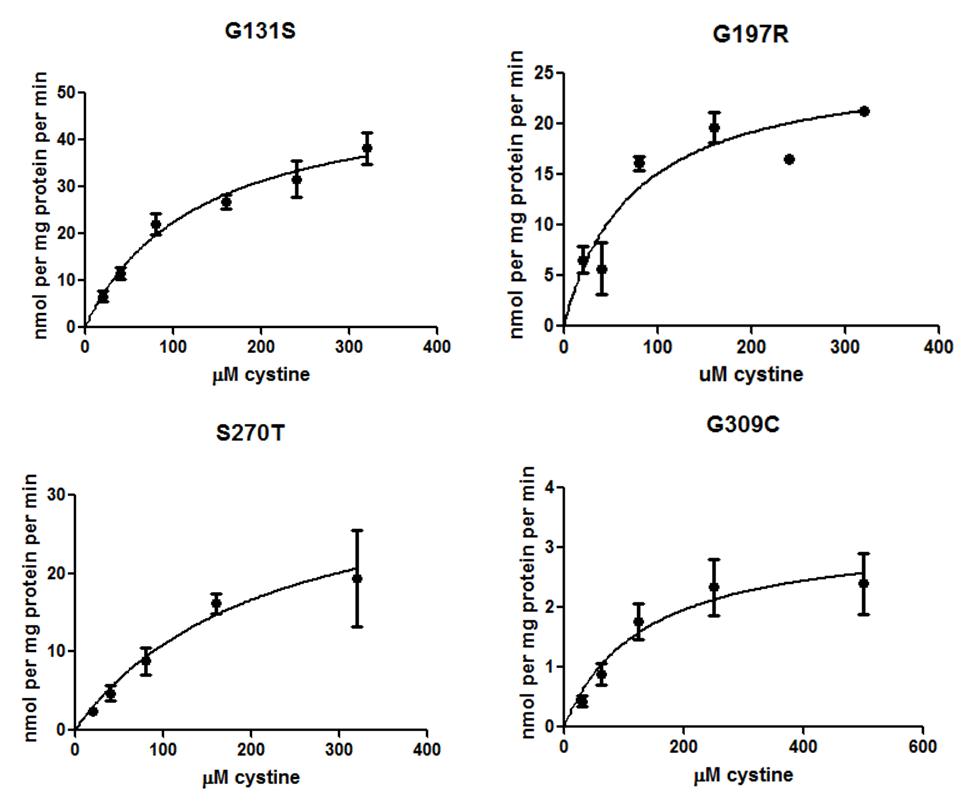
**

**Fig.S6 Determination of kinetic parameters for CTNSΔ-gydql and gain-of-function mutants**

The average *K*m (μM) and *V*max (nmol of cystine⋅mg⋅protein^-1^min^-1^) values were determined by non-linear regression analysis of *V* compared with [S] graphs showing saturation kinetics using GraphPad Prism Version 5.1 software. The experiment was repeated a minimum of two times for each test construct in duplicates for each cystine concentration (± S.D. n=4).


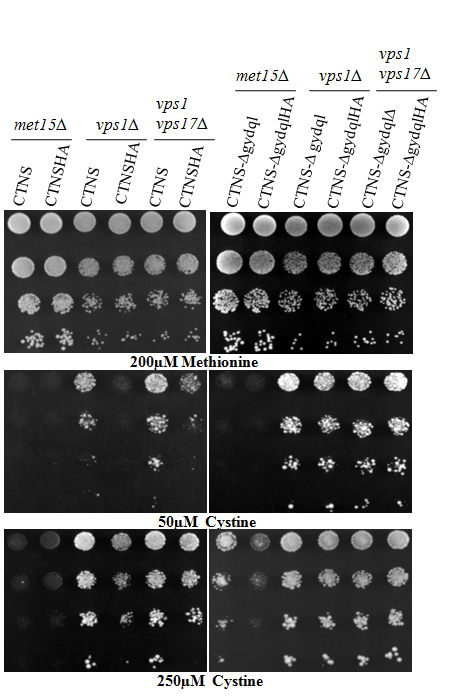


**Fig.S7 Functional analysis of TEF-CTNS, TEF-CTNSHA and TEF-CTNS-Δgydql, TEF-CTNS-Δ gydqlHA constructs transformed in different vacuolar protein sorting defective mutants (single and double delete) .**

Different vacuolar protein sorting defective mutants were transformed with, tagged (HA tag) and untagged CTNS, or CTNS-Δgydql constructs expressed under the TEF promoter, and examined by dilution spotting on minimal media containing 200µM Methionine (control plates), and different concentration of cystine .
